# Inhibition of mitochondrial complex III or dihydroorotate dehydrogenase (DHODH) triggers formation of poly(A)^+^ RNA foci adjacent to nuclear speckles following activation of ATM (ataxia telangiectasia mutated)

**DOI:** 10.1080/15476286.2022.2146919

**Published:** 2022-11-22

**Authors:** Shuntaro Miyake, Seiji Masuda

**Affiliations:** a Division of Integrated Life Sciences, Graduate School of Biostudies, Kyoto University, Kyoto, Japan; b Department of Food Science and Nutrition, Faculty of Agriculture, Kindai University, Nara, Japan; c Agricultural Technology and Innovation Research Institute, Kindai University, Nara, Japan; d Antiaging Center, Kindai University, Higashiosaka, Japan

**Keywords:** Poly(A)^+^ RNA, dihydroorotate dehydrogenase (DHODH), ataxia telangiectasia mutated (ATM), pyrimidine, respiratory chain complex III

## Abstract

Intracellular and intercellular signalling networks play an essential role in optimizing cellular homoeostasis and are thought to be partly reflected in nuclear mRNA dynamics. However, the regulation of nuclear mRNA dynamics by intracellular and intercellular signals remains largely unexplored, and research tools are lacking. Through an original screening based on the mRNA metabolic mechanism, we discovered that eight well-known inhibitors cause significant nuclear poly(A)^+^ RNA accumulation. Among these inhibitors, we discovered a new mRNA metabolic response in which the addition of antimycin A, an inhibitor of mitochondrial respiratory-chain complex III (complex III), resulted in a marked accumulation of poly(A)^+^ RNA near the nuclear speckles. Furthermore, dihydroorotate dehydrogenase (DHODH) inhibitors, a rate-limiting enzyme in the intracellular *de novo* pyrimidine synthesis reaction that specifically exchanges electrons with complex III, also caused a remarkable accumulation of nuclear poly(A)^+^ RNA adjacent to the nuclear speckles, which was abolished by extracellular uridine supply, indicating that the depletion of intracellular pyrimidine affects poly(A)^+^ RNA metabolism. Further analysis revealed that ataxia telangiectasia mutated (ATM), a serine and threonine kinase and a master regulator of DNA double-strand break (DSB) and nucleolar stress, is required for this poly(A)^+^ RNA nuclear accumulation phenomenon. This study reports new insights into novel aspects of nuclear poly(A)^+^ RNA metabolism, especially the relationship between mitochondrial respiratory-chain functions, pyrimidine metabolism, and nuclear RNA metabolism.

## Introduction

In eukaryotes, mRNA is first transcribed as a precursor mRNA and undergoes several processings, such as 5′ capping, splicing, and 3′ polyadenylation. These processes are mediated by several ribonucleoproteins and are tightly regulated to ensure that correctly matured mRNAs are facilitated to be exported from the nucleus. However, many aspects of this sophisticated regulation of mRNA processing remain unelucidated [1–3]. Recently, the regulatory mechanism of mRNA splicing in the nucleus has been elucidated using splicing inhibitors, revealing that spliceostatin A, pladienolide B, Gex1A, and H3B-7700 inhibit splicing by binding to SF3B1, a component of U2 small nuclear RNA (U2snRNP), which is a spliceosomal factor, contributing to the understanding of splicing regulation [4–7]. Additionally, aberrant splicing has been reported as the pathogenesis of various diseases, including cancer [8].

Various external signals are altered by specific molecules in the cell and transmitted into the cell, called the signal transduction pathway, and influences the transmission of information in and out of the cell, optimizing cellular responses and regulating cell proliferation and differentiation [9]. Some of these signals are transmitted to the nucleus and trigger gene expression, suggesting that in the nucleus, other signals elicit dynamic changes in mRNA metabolism. However, signal transduction-dependent changes in nuclear mRNA metabolism remain unelucidated.

In our previous studies, we established a screening method for inhibitors of nuclear mRNA metabolism by focusing on poly(A)^+^ RNA metabolism [10,11], and by exploring food-derived components, we discovered that apigenin and luteolin have splicing–inhibitory activity [12]. Using this technique, an inhibitor with a known and well-defined inhibitory target was added to cells, and changes in mRNA metabolism can be observed as an accumulation of nuclear poly(A)^+^ RNA. Herein, we aimed to search for a known inhibitor that affects nuclear mRNA metabolism and to discover new signals that alter nuclear mRNA metabolism and tools to study them.

Dihydroorotate dehydrogenase (DHODH) is an enzyme responsible for the rate-limiting step in the fourth step of the cellular *de novo* pyrimidine synthesis reaction, the conversion of dihydroorotate (DHO) to orotate (ORO). In higher eukaryotes, it is localized to the outer surface of the inner mitochondrial membrane. In the inner mitochondrial membrane, mitochondrial respiratory-chain complexes transfer electrons from electron donors to electron acceptors via redox reactions, and DHODH is linked by a reaction that converts the electron acceptor coenzyme q (CoQ) to CoQH2, a substrate of respiratory-chain complex III [13,14]. DHODH inhibitors have therapeutic effects against several diseases, such as malaria, autoimmune diseases, cancer, and myeloid malignancies [15–18]. Additionally, they have been reported to have antiviral activity against various viruses, such as flavivirus, ebola virus, herpes virus, enterovirus, and influenza A and B viruses [19–23]. Interestingly, DHODH inhibitors are also expected to be effective as therapeutic agents against SARS-CoV-2, which has recently caused a pandemic [24,25], making it increasingly crucial to elucidate its effects on cells.

In this study, we show for the first time that eight of the 362 known inhibitors cause nuclear poly(A)^+^ RNA accumulation. First, among the active inhibitors, the mitochondrial respiratory-chain complex III inhibitor accumulated poly(A)^+^ RNA adjacent to nuclear speckles by inhibiting DHODH that exchanges electrons. Second, this poly(A)^+^ RNA nuclear accumulation was abolished by extracellular uridine addition, suggesting that the depletion of pyrimidine promotes nuclear accumulation of poly(A)^+^ RNA. Third, the activation of ataxia telangiectasia mutated (ATM), a serine and threonine kinase and a master regulator of DNA double-strand break (DSB) and nucleolar stress, is required for this poly(A)^+^ RNA accumulation. Briefly, by focusing on nuclear poly(A)^+^ RNA metabolism and re-evaluating known inhibitors, we discovered a new nuclear poly(A)^+^ RNA regulatory pathway and reported a new link between pyrimidine and nuclear poly(A)^+^ RNA metabolism regulation for the first time.

## Results

### Screening of inhibitors of perturbing nuclear poly(A)^+^ RNA metabolism by the RNA–fluorescence in situ hybridization (RNA-FISH)

To search for inhibitors that affect mRNA metabolism, we first screened 362 known inhibitors with our established method using the RNA–fluorescence *in situ* hybridization (RNA-FISH) with Alexa594-labelled oligo dT45 probe [10,11]. To examine an inhibitor with perturbing nuclear mRNA metabolism, we used the Screening Committee of Anticancer Drugs (SCADS) inhibitor kits I–IV, which cover known inhibitors of various intracellular signals. Each inhibitor was uniformly added to U2OS cells at 2 μM. After 24 h of incubation, the localization of poly(A)^+^ RNA in the cell was observed by RNA-FISH. Cell images were quantified by taking the nuclear/whole-cell ratio of poly(A)^+^ RNA signals probed with Alexa594labelled oligo dT45. The solvent was used as a negative control, and Gex1A, a well-known splicing inhibitor, was used as a positive control.

**Figure 1.**
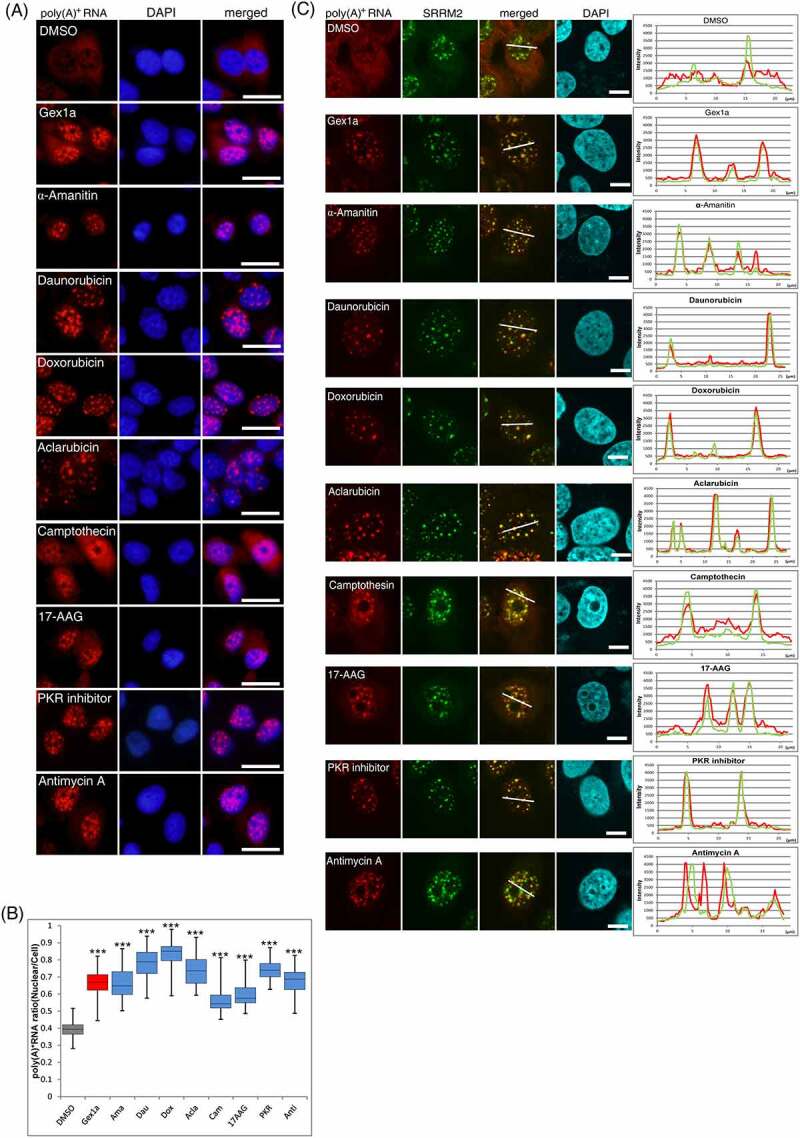
Screening of inhibitors for perturbing nuclear poly(A)^+^ RNA metabolism.

Screening results showed that among 362 inhibitors, eight of them, namely, daunorubicin, doxorubicin, aclarubicin, α-amanitin, camptothecin, 17-AAG, PKR inhibitor, and antimycin A, gave a significant nuclear poly(A)^+^ RNA accumulation phenotype (Fig. 1A, B). To investigate the localization of poly(A)^+^ RNA in the nucleus, we performed the costaining of poly(A)^+^ RNA and SRRM2 (serine/arginine repetitive matrix 2), a marker of nuclear speckles, in inhibitor-treated cells because poly(A)^+^ RNA accumulated in the speckles when mRNA splicing was inhibited [4,26]. Confocal microscopy revealed that poly(A)^+^ RNA was colocalized with the nuclear speckles in GEX1A-treated positive control cells and seven of the eight inhibitor-treated cells, raising the possibility that these inhibitors may have influenced mRNA splicing. In contrast, antimycin A treatment accumulated poly(A)^+^ RNA adjacent to nuclear speckles (Fig. 1C). We further investigated the localization of the antimycin A-induced nuclear poly(A)^+^ RNA foci using another component of the speckles, MALAT1 [27], a well-known lncRNA and a representative marker of nuclear speckles. The localization of poly(A)^+^ RNAs foci in the nucleus was not colocalized with MALAT1 (Supplemental Figure 1A). These results may suggest that antimycin A, an inhibitor of mitochondrial respiratory-chain complex III, affected metabolic processes which are distinct from mRNA splicing.

(A) Screening of inhibitors that affect nuclear poly(A)^+^ RNA metabolism. RNA-FISH was performed to determine the localization of bulk poly(A)^+^ RNA. U2OS cells were treated with 2 µM of each compound for 24 h. GEX1A (30 ng/mL) was used as a positive control. Bulk poly(A)^+^ RNA was visualized with Alexa Fluor 594-labelled oligo-dT_45_ probe. The nuclei were visualized with DAPI. Scale bar, 50 μm. (B) The ratio of the nuclear distribution of poly(A)^+^ RNA was analyzed. Signal intensities of the whole cell and the nucleus were quantified using ImageJ (n = 40). Boxes indicate median (centerline) and upper and lower quartiles. Whiskers indicate the lowest and highest values. Statistical analysis was performed using one-way analysis of variance (ANOVA) followed by Dunnett’s test. ***p < 0.001. Dau: daunorubicin, Dox: doxorubicin, Acla: aclarubicin, Ama: α-amanitin, Cam: camptothecin, Anti: antimycin (C) Localization of bulk poly(A)^+^ RNA and nuclear speckles. Poly(A)^+^ RNA (red), speckle (green), and chromosomal DNA (blue) were visualized in U2OS cells. The cells were treated with each inhibitor for 24 h. Scale bar, 10 μm. Signal intensities on the white line for poly(A)^+^ RNA and speckle are plotted and presented with red and green lines in the right panel, respectively.

Subsequently, we immuno-stained paraspeckles, which are nuclear structures located in the vicinity of speckles, using their marker, PSP1, a representative paraspeckle protein, and NEAT1 [28], a well-known lncRNA. However, poly(A)^+^ RNA foci were not also colocalized with paraspeckles (Supplemental Figure 1B, C). Furthermore, accumulated poly(A)^+^ RNA foci treated with antimycin A were not colocalized with Cajal body [based on coilin staining] (Supplementary Figure 1D). Additionally, antimycin A increased the nucleolar localization of coilin, suggesting the occurrence of nucleolar stress [29]. Then, we examined whether antimycin A induced nuclear poly(A)^+^ RNA accumulation in other cell types. Antimycin A also induced nuclear poly(A)^+^ RNA accumulation in HeLa, MCF7, and U2OS cells, from 0.125 to 2 μM at 12, 24, and 36 h (Supplemental Figure 2A, B, C). Among the above-mentioned time courses in all cell lines, nuclear poly(A)^+^ RNA accumulation was most markedly observed at 36 h.

This inhibitor screening identified eight inhibitors that altered poly(A)^+^ RNA metabolism. Seven of these are supposed to regulate mRNA splicing, either directly or indirectly. In contrast, antimycin A appeared to act through an unknown pathway in nuclear poly(A)^+^ RNA metabolism. This is the first report on poly(A)^+^ RNA accumulation due to inhibition of mitochondrial respiratory chain III. Therefore, we focused our study on the mechanism of this novel observation.

### Inhibition of de novo pyrimidine synthesis perturbs poly(A)^+^ RNA metabolism

**Figure 2.**
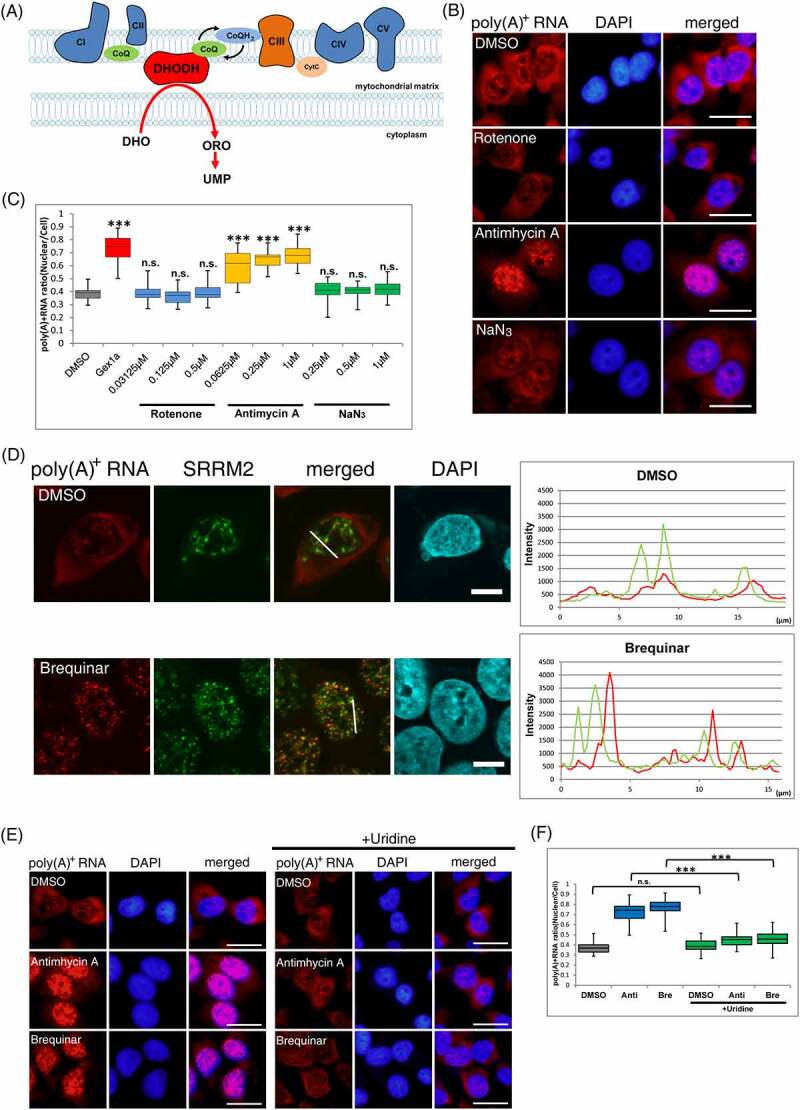
*De novo* pyrimidine synthesis pathway perturbs poly(A)^+^ RNA metabolism.

Mitochondrial respiratory-chain complexes are located in the inner mitochondrial membrane and are responsible for intracellular adenosine triphosphate (ATP) production by exchanging electrons in the order I to V [30] (Fig. 2A). Since the inhibition of respiratory-chain complex III led to the accumulation of poly(A)^+^ RNA in unidentified foci in the nucleus, another respiratory-chain inhibition was investigated to determine whether the inhibition of mitochondrial electron transfer was crucial for nuclear poly(A)^+^ RNA foci formation. When rotenone, a respiratory chain complex I inhibitor, and NaN3, a complex IV inhibitor, were added to MCF7 cells, no nuclear accumulation of poly(A)^+^ RNA was observed (Fig. 2B). Moreover, another complex III inhibitor, atovaquone, which differs from antimycin A in both structure and mechanism of action [31], induced poly(A)^+^ RNA accumulation similar to antimycin A, and the localization of poly(A)^+^ RNA was not colocalized with nuclear speckles (Supplemental Figure 3A, B), suggesting that the nuclear poly(A)^+^ RNA accumulation was specific for respiratory-chain complex III repression.

(A) Schematic of mitochondrial respiratory-chain complexes and DHODH. CI–V: mitochondrial complex I–V, CoQ: CoenzymeQ, CytC: cytochrome c, DHO: dihydroorotic acid, ORO: orotic acid, UMP: uridine monophosphate, (B) Representative localization of poly(A)^+^ RNA in MCF7 cells treated with rotenone (0.5 µM), antimycin A (0.25 µM), and NaN_3_ (1 µM) for 36 h. The nuclei were visualized with DAPI. Scale bar, 50 μm. (C) The ratio of the nuclear distribution of poly(A)^+^ RNA was analyzed. Signal intensities of the whole cell and the nucleus were quantified using ImageJ (n = 40). Boxes indicate median (centerline) as well as upper and lower quartiles. Whiskers indicate the lowest and highest values. Statistical analysis was performed using one-way ANOVA followed by Dunnett’s test. ***p < 0.001. (D) The localization of bulk poly(A)^+^ RNA and nuclear speckles. Poly(A)^+^ RNA (red), speckles (green), and chromosomal DNA (blue) were visualized for colocalization analysis in MCF7 cells. The cells were treated with DMSO or brequinar (0.5µM) for 36 h. Scale bar, 10 μm. Signal intensities on the white line are plotted in the right panels. Poly(A)^+^ RNA and speckle signals are shown with red and green lines, respectively. (E) RNA-FISH was performed to determine the localization of bulk poly(A)^+^ RNA. MCF7 cells were treated with DMSO, antimycin A (0.25 µM), or brequinar (0.5 µM), with or without uridine 2 mM for 36 h. The bulk poly(A)^+^ RNA was visualized with Alexa Fluor 594-labelled oligo-dT_45_ probe. The nuclei were visualized with DAPI. Scale bar, 50 μm. Rote: rotenone, Anti: antimycin A, Bre: brequinar (F) The ratio of the nuclear distribution of poly(A)^+^ RNA was analyzed. Signal intensities of the whole cell and the nucleus were quantified using ImageJ (n = 40). Boxes show median (centerline) as well as upper and lower quartiles. Whiskers show the lowest and highest values. Statistical analysis was performed using one-way ANOVA followed by Tukey’s test. ***p < 0.001. Rote: rotenone, Anti: antimycin A, Bre: brequinar

Studies have shown that respiratory-chain complex III exchanges electrons with the electron transfer system and DHODH [14]. Therefore, inhibition of the respiratory-chain complex III decreased DHODH activity [32]. To examine whether the accumulation of poly(A)^+^ RNA in the nucleus by inhibitors of respiratory-chain complex III is due to the inhibition of DHODH activity rather than electron transfer in the respiratory chain, we used brequinar, a specific DHODH inhibitor. A significant nuclear accumulation of poly(A)^+^ RNA was observed upon the brequinar addition. Similar to the addition of respiratory-chain complex III inhibitors, poly(A)^+^ RNA accumulation by brequinar was not colocalized with nuclear speckles (Fig. 2C, D).

Additionally, the accumulation of nuclear poly(A)^+^ RNA was observed with teriflunomide, another DHODH inhibitor with a different chemical structure than brequinar [33]. Similarly, with antimycin A and brequinar, the poly(A)^+^ RNA foci did not colocalize with nuclear speckles (Supplemental Figure 3A, B, C), indicating that nuclear poly(A)^+^ RNA accumulation was due to DHODH inhibition through electron transfer from complex III rather than respiratory-chain inhibition. To confirm this, cellular DHODH activity was measured using a previously reported method [34]. All inhibitors used in this study reduced the DHODH activity (Supplemental Figure 3D), although the reduction rate by each inhibitor was from 40% to 60%. This suggests that the accumulation of poly(A)^+^ RNA in the nucleus was triggered by the inhibition of *de novo* pyrimidine synthesis. Therefore, an excess amount of uracil was added to the medium along with antimycin A or brequinar because, in addition to the *de novo* synthesis of the pyrimidine pathway, pyrimidine is synthesized by the extracellular uptake of uracil using a salvage pathway (Fig. 2A) [13]. The addition of uracil fully restored the nuclear accumulation of poly(A)^+^ RNA by antimycin A and brequinar treatment (Fig. 2D, E), indicating that nuclear poly(A)^+^ RNA accumulation by antimycin A and brequinar was not due to mitochondrial respiratory-chain inhibition but further supporting that it is induced by *de novo* pyrimidine synthesis inhibition.

These results suggest that nuclear poly(A)^+^ RNA accumulation is induced in response to a decrease in intracellular pyrimidine concentration. Therefore, we investigated whether the nuclear accumulation of poly(A)^+^ RNAs can be observed by inhibiting purine synthesis. To investigate this, we added methotrexate, an inhibitor of folate metabolism and a typical inducer of purine synthesis inhibition [35], to MCF7 cells and observed poly(A)^+^ RNA localization in the cell by RNA-FISH. However, treatment with methotrexate did not cause poly(A)^+^ RNA accumulation in the nucleus (Supplemental Figure 3A, B), suggesting that nuclear poly(A)^+^ RNA accumulation is specific to a decrease in pyrimidine synthesis.

### Regulation of respiratory chain complex III and DHODH inhibition-mediated nuclear accumulation of poly(A)^+^ RNA by ATM

**Figure 3.**
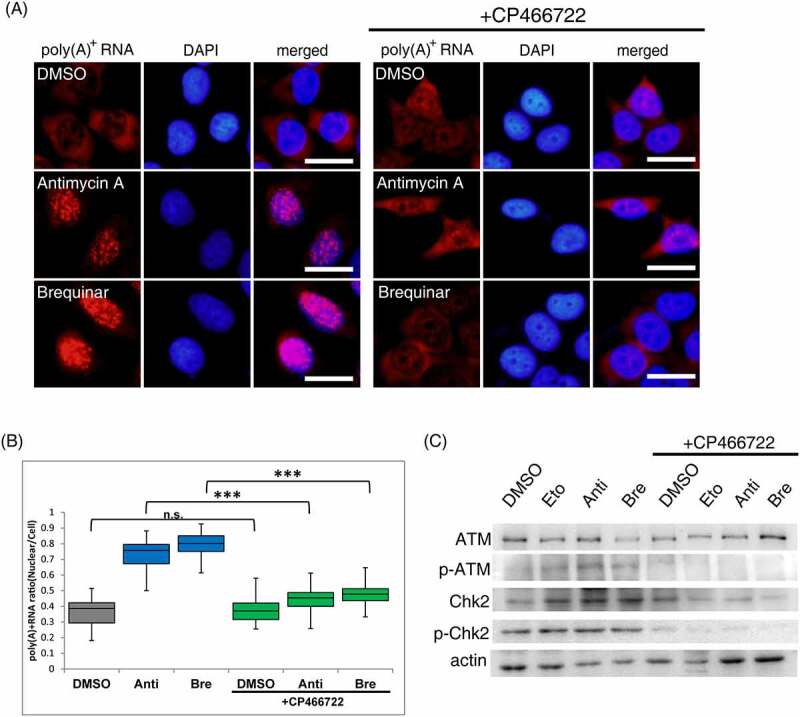
ATM activation is required to elicit nuclear accumulation of poly(A)^+^ RNA by inhibitors of the respiratory chain complex III and DHODH.

Next, the mechanism leading from DHODH inhibition to the accumulation of poly(A)^+^ RNA in the nucleus was investigated. Studies have shown that DHODH inhibition causes DNA damage and nucleolar stress [36–39]. Common to DNA damage and nucleolar stress, ATM, as a master regulator, is activated and phosphorylates downstream factors [40]. It has also been reported to be activated by brequinar to evoke downstream immune factor responses [20], and p53, a typical downstream factor of ATM, is activated by DHODH inhibitors [32,41]. Based on these reports, we hypothesize that the accumulation of nuclear poly(A)^+^ RNA due to DHODH inhibition is mediated by ATM. To test this possibility, we investigated whether blocking the signal from ATM could eliminate the nuclear accumulation of poly(A)^+^ RNA by antimycin A and brequinar. Antimycin A or brequinar addition accumulated poly(A)^+^ RNA in the nucleus (Fig. 3A, B). In contrast, CP466722, as the ATM inhibitor [42], addition combined with antimycin A and brequinar abolished the accumulation of poly(A)^+^ RNA in the nucleus. Inhibition of ATM activity was confirmed by Western blotting of ATM autophosphorylation and phosphorylation of Chk2, the primary target of ATM phosphorylation (Fig. 3C). Etoposide, a topoisomerase II inhibitor, was used as a positive control to activate ATM [43,44]. ATR (ataxia telangiectasia and Rad3-related), one of the phosphoinositide 3-kinase-related kinases (PIKK) like ATM, also phosphorylates serine and threonine in response to DNA damage [45]. To determine whether ATM activation plays a crucial role in nuclear poly(A)^+^ RNA accumulation, we also investigated whether ATR plays a role in poly(A)^+^ RNA accumulation in the nucleus. The nuclear poly(A)^+^ RNA accumulation was partially abolished when VE822, a specific ATR inhibitor [46], was simultaneously added with antimycin A and brequinar (Supplemental Figure 4A, B). Thus, these results indicate that ATM activation is predominantly required for nuclear poly(A)^+^ RNA accumulation.

(A) RNA-FISH was performed to determine the localization of bulk poly(A)^+^ RNA. MCF7 cells were treated with DMSO, antimycin A (0.25 µM), or brequinar (0.5 µM) with or without CP466722 (20 µM) for 36 h. The bulk poly(A)^+^ RNA was visualized by Alexa Fluor 594-labelled oligo-dT45 probe. The nuclei were visualized with DAPI. Scale bar, 50 μm. (B) The ratio of the nuclear distribution of poly(A)^+^ RNA was analyzed. Signal intensities of the whole cell and the nucleus were quantified using ImageJ (n = 40). Boxes indicate median (centerline) as well as upper and lower quartiles. Whiskers indicate the lowest and highest values. Statistical analysis was performed using one-way ANOVA followed by Tukey’s test. ***p < 0.001. (C) Protein expression of ATM, p-ATM, Chk2, and p-Chk2 were examined by immunoblotting. Actin was used as a loading control. Anti: antimycin A, Bre: brequinar

### DHODH inhibition causes relocalization of nucleolar components

**Figure 4.**
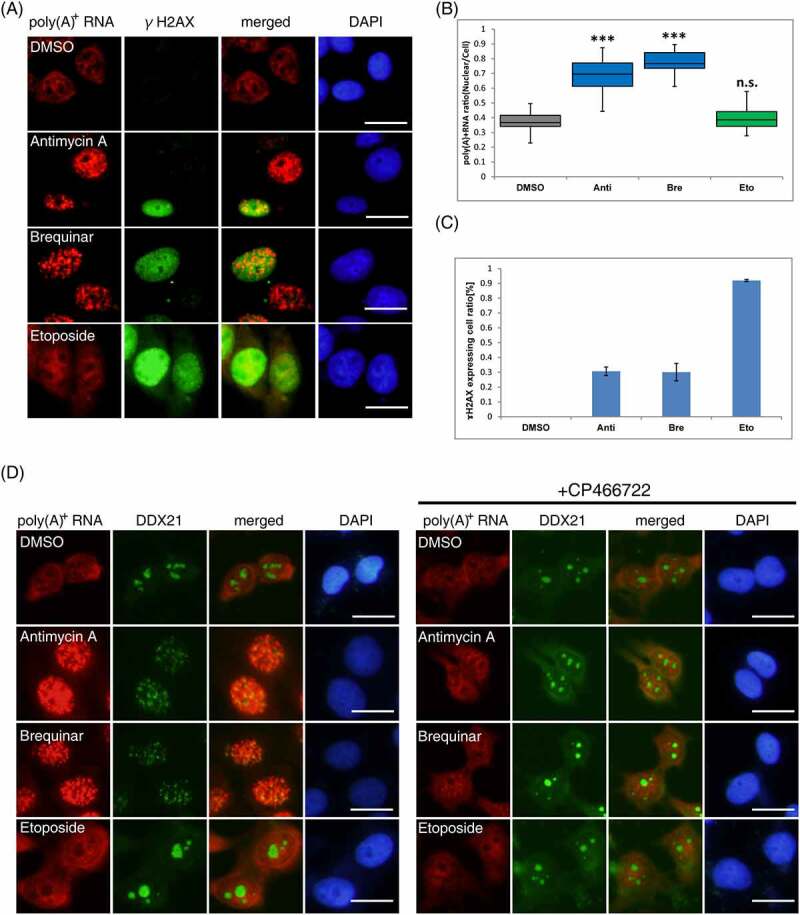
Relocalization of the nucleolar component by DHODH inhibition.

DHODH inhibition causes DNA damage and nucleolar stress [36–39], which activate ATM kinase, raising the possibility that DNA damage and nucleolar stress are the direct causes of nuclear poly(A)^+^ RNA accumulation. Therefore, we first observed γH2AX [47], a representative DNA damage marker, to examine this possibility. In cells with antimycin A or brequinar, γH2AX was observed in approximately 30% of cells where nuclear poly(A)^+^ RNA accumulation occurred (Fig. 4A, B, C). In contrast, in etoposide-treated cells, a positive control that induces DSB, γH2AX activation was observed in almost all cells, but no nuclear accumulation of poly(A)^+^ RNA was observed (Fig. 4D). These results indicate that DNA damage is not associated with nuclear poly(A)^+^ RNA accumulation and that ATM activation alone is insufficient for poly(A)^+^ RNA accumulation by DHODH inhibition.

(A) The localization of bulk poly(A)^+^ RNA and ɤH2AX. Poly(A)^+^ RNA (red), ɤH2AX (green), and chromosomal DNA (blue) were visualized in MCF7 cells. Cells were treated with DMSO, antimycin A (0.25 µM), brequinar (0.5 µM), or etoposide (5 µM) for 36 h. Scale bar, 50 μm. (B) The ratio of the nuclear distribution of poly(A)^+^ RNA was analyzed. The signal intensities of the whole cell and the nucleus were quantified using ImageJ (n = 40). Boxes show median (centerline) as well as upper and lower quartiles. Whiskers show the lowest and highest values. Statistical analysis was performed using one-way ANOVA followed by Dunnett’s test. ***p < 0.001. (C) The ratio of the ɤH2AX-expressing cells was counted (n = 300). Each value is the mean ± SD of three independent experiments. (D) The localization of bulk poly(A)^+^ RNA and DDX21. Poly(A)^+^ RNA (red), DDX21 (green), and chromosomal DNA (blue) were visualized in MCF7 cells. Cells were treated with DMSO, antimycin A (0.25 µM), brequinar (0.5 µM), or etoposide (5 µM) with or without CP466722 (20 µM) for 36 h. The bulk poly(A)^+^ RNA was visualized by Alexa Fluor 594-labelled oligo-dT45 probe. The nuclei were visualized with DAPI. Scale bar, 50 μm. Anti: antimycin A, Bre: brequinar, Eto: etoposide

Next, we examined nucleolar stress caused by antimycin A and brequinar. Recently, there are some reports that DHODH inhibition resulted in nucleolar stress [38,39] but there are only few reports on nucleolar-related factors that affect poly (A)^+^ RNA metabolism in the nucleus by DHODH inhibitors. A study reported that in zebrafish neurons and human melanoma cells, the pol I-binding RNA helicase, DDX21, was localized from the nucleolus to the nucleoplasm by DHODH inhibitors and increased its binding to mRNA [48]. Then, we speculated that capturing poly(A)^+^ RNA by relocalized DDX21 may result in the manifestation of the poly(A)^+^ RNA foci in the nucleus. To examine this, the localization of DDX21 and nucleolin [49], a major component of the nucleolus, was observed in cells treated with antimycin A or brequinar. Immunostaining for each factor revealed that DDX21 and nucleolin were relocalized from the nucleolus to the nucleoplasm by adding antimycin A or brequinar (Fig. 4D, Supplemental Figure 5A). The addition of CP466722 combined with antimycin A or brequinar inhibit the relocalization of DDX21 and nucleolin. In the etoposide-treated cells, DDX21 and nucleolin did not relocalize. Contrary to our expectations, confocal microscopic observations showed that each factor did not colocalize with the accumulated poly(A)^+^ RNA (Supplemental Figure 5B, C).

To further investigate the association between nuclear poly (A)^+^ RNA accumulation and nucleolar stress by DHODH inhibitors, we subsequently examined whether TCOF1, a factor that functions with RNA polymerase I in the nucleolus, was involved in poly (A)^+^ RNA accumulation. We opted for TCOF1 because it is one of the main upstream targets for phosphorylation by ATM in the rDNA damage response [50,51]. In addition, a previous study reported that dysfunctional TCOF1 causes changes in the localization of DDX21 to the nucleoplasm [52]. Therefore, we investigated to ascertain if the accumulation of nuclear poly(A)^+^ RNA triggered by DHODH inhibitors was due to TCOF1-mediated nucleolar stress. In control cells, TCOF1 was localized in the nucleolus (Supplemental Figure 6A). In contrast, it was relocalized to the nucleolar caps of brequinar-treated cells. To examine the relationship between TCOF1 expression and nuclear accumulation of poly(A)^+^ RNA, we transfected siRNA (small interfering RNA) against TCOF1 with or without brequinar. Significant nuclear accumulation of poly(A)^+^ RNA was observed even in cells without TCOF1 expression (Supplemental Figure 6B). These results indicate that ATM-mediated changes in the localization of nucleolar components are induced by antimycin A and brequinar; however, DDX21 and TCOF1, which are key molecules in nucleolar stress, are not involved in the nuclear poly(A)^+^ RNA accumulation by these inhibitors, i.e. antimycin A and brequinar.

As for the relationship between nucleolar stress and nuclear poly(A)^+^ RNA metabolism, it has been previously reported that actinomycin D, an inhibitor of RNA polymerase I, which causes nucleolar stress, localizes poly(A)^+^ RNA adjacent to nuclear speckles [53]. In our experimental system, we also observed the accumulation of poly(A)^+^ RNA (Supplemental Figure 6D), but the detailed mechanism of this occurrence has not yet been elucidated. One of the most actively transcribed nucleic acid is rRNA in the nucleolus. Thus, it may function as a sensor of pyrimidine deficiency. Taken together, the accumulation of nuclear poly(A)^+^ RNA by DHODH inhibition not only requires ATM activation but also involves unidentified signals that require further analyses.

## Discussion

Nuclear mRNA metabolism is tightly regulated by various signals inside and outside the cell and is essential for regulating gene expression. However, the intracellular pathways that regulate nuclear mRNA metabolism are largely unexplored, and elucidation of these pathways as well as creation of research tools will become increasingly important. In this study, eight inhibitors were found to cause nuclear poly(A)^+^ RNA accumulation. Seven of these inhibitors, namely, daunorubicin, doxorubicin, aclarubicin, camptothecin, 17AAG, α-amanitin, and PKR inhibitor, showed that poly(A)^+^ RNA was accumulated in the nuclear speckle. The treatment of splicing inhibitors, such as GEX1A, accumulated poly(A)^+^ RNA in the nuclear speckle [12], implying that the seven inhibitors stated above probably affected splicing. A detailed search for previous studies revealed that several compounds, namely, camptothecin, 17AAG, α-amanitin, and PKR inhibitors, are associated with splicing [54–63].

Camptothecin is a quinoline alkaloid used as an inhibitor of topoisomerase I, and its analogues are used in cancer chemotherapy [54]. Its derivatives, including topotecan, have been reported to bind to NHP2L1 and U4 snRNA, which form spliceosomes [55], indicating that splicing is affected by camptothecin derivatives. Furthermore, 17AAG is an inhibitor of heat shock protein 90 (HSP90), a molecular chaperone responsible for heat shock [56]. RNA sequence analysis has revealed that the HSP90 inhibitor, onalespib, alters mRNA splicing [57], suggesting that HSP90 inhibition by 17AAG also causes splicing inhibition. α-amanitin is a cyclic peptide of eight amino acids that inhibits RNA transcription by binding to RNA polymerase II [58]. Its treatment concentrates snRNPs and SR proteins in speckles, and accumulation of these splicing factors could reduce splicing efficiency [59–61]. PKR is an RNA-dependent protein kinase that inhibits transcription by phosphorylating cytoplasmic eukaryotic initiation factor 2α (eIF2α) in the cytoplasm [62]. More recently, PKR has been reported to be involved in the spliceosome formation of globin mRNA, and its depletion or treatment with PKR inhibitors inhibits the splicing of globin pre-mRNA [63]. Daunorubicin, doxorubicin, and aclarubicin are multifunctional compounds belonging to the anthracycline family, which are anticancer drugs widely used in clinical practice [64,65]. The most well-established antitumor mechanism of action of anthracyclines is DNA DSBs via topoisomerase II inhibition [66]. Etoposide, another topoisomerase II inhibitor, did not cause nuclear poly(A)^+^ RNA accumulation in this study, indicating that DNA DSBs evoked by topoisomerase II inhibitors did not directly induce nuclear poly(A)^+^ RNA accumulation. Additionally, recent reports have shown that anthracyclines affect splicing, but a reliable pathway for their mechanism of action remains unclear [67,68].

Further studies are essentially required to determine why these inhibitors affect splicing. So far, our screening system, even with a limited library, has effectively selected compounds that have been suggested to affect nuclear mRNA metabolism by previous studies. Moreover, only 2 μM of each inhibitor was examined to observe the change in nuclear mRNA localization owing to the limited amount of inhibitors available, possibly missing inhibitors that alter nuclear mRNA localization. Thus, it is highly anticipated that a larger library size and changes in the concentrations used will allow for a more efficient search for inhibitors that regulate mRNA metabolism.

The inhibitors of respiratory-chain complex III found in this study and specific DHODH inhibitors showed significant nuclear accumulation of poly(A)^+^ RNA in the vicinity of the nuclear speckles. This poly(A)^+^ RNA accumulation in the nucleus was abolished following external uridine addition, indicating that the inhibition of intracellular pyrimidine synthesis causes poly(A)^+^ RNA accumulation in the nucleus. Methotrexate addition, a typical inhibitor of purine synthesis, did not cause nuclear poly(A)^+^ RNA accumulation, suggesting that the accumulation of nuclear poly(A)^+^ RNA by inhibiting respiratory-chain complex III and DHODH is not a simple nucleic acid deficiency but a specific effect of pyrimidine reduction. Speculating on the relationship between pyrimidine reduction and nuclear poly(A)^+^ RNA accumulation, it is likely that reactions specific to pyrimidine rather than purine syntheses are involved, one of which is the UDP-glycosylation reaction. UDP-sugars, such as UDP-GlcNAc, GalNAc, and glucose, are donor substrates for glycosylation reactions on proteins [69–71]. These glycosylation reactions are essential for cellular functions as post-transcriptional regulation of various proteins. O-GlcNAcylation by the UDP-GlcNAc transferase, OGT (O-GlcNAc transferase), regulates various intracellular signals, including mRNA metabolic factors, such as RNA pol II [72]. In stem cells, the nuclear translocation of the transcription factor, SOX2, is regulated by the UDP-GlcNAc transferase, and these reactions are interfered by DHODH inhibitors [73]. Thus, an attractive possibility is that DHODH inhibition alters posttranslational protein modification with UDP-glycosylation and affects mRNA metabolism.

The pathway from DHODH inhibition to nuclear poly(A)^+^ RNA accumulation involves ATM activation. Although DNA DSBs also activate ATM, they do not cause nuclear poly(A)^+^ RNA accumulation, suggesting that ATM activation is required but insufficient. Thus, another intracellular signal is required to elicit the nuclear poly(A)^+^ RNA accumulation. We also observed changes in the localization of nucleolar components in cells with antimycin A or brequinar treatment. This observation implies that p53 stress is provoked by antimycin A or brequinar and raises the possibility that nucleolar stress, as another signal, may become the cause of nuclear poly(A)^+^ RNA accumulation. To further investigate the relationship between nucleolar stress and poly(A)^+^ RNA accumulation, we dealt with DDX21 and TCOF1. The treatment with antimycin A and brequinar did not colocalize the poly(A)^+^ RNA foci with DDX21, implying that DDX21 seemed unrelated to poly(A)^+^ RNA foci formation. The same was true for TCOF1. Therefore, expanding the screening scale may help find another intracellular signal.

Recent studies have highlighted DHODH inhibitors as potential anti-immunity, antiviral, anticancer, and acute myeloid leukaemia therapeutic targets [74–77]. Upon their treatment, nuclear poly(A)^+^ RNA accumulation might be the key to eliciting these phenomena. Although many DHODH inhibitors have been identified in recent decades, only leflunomide and teriflunomide have received the United States Food and Drug Administration approval, and DHODH inhibitors are still not widely used despite their potential. Because DHODH inhibitors evoke ATM activation and nuclear poly(A)^+^ RNA accumulation as well as induce nucleolar stress, the pyrimidine synthesis pathway may play a key role in maintaining nuclear homoeostasis. Therefore, evaluating the function of DHODH inhibitors plays a crucial role in developing more effective therapeutics. Thus, we hope that the results of this study will be helpful in developing therapeutic agents and elucidating mRNA metabolism.

## Materials and methods

### Cell culture

U2OS, HeLa, and MCF7 cells were maintained in Dulbecco’s Modified Eagle Medium (FUJIFILM Wako Pure Chemical Corporation, Osaka, Japan) supplemented with 6% heat-inactivated foetal bovine serum at 37°C.

### Antibodies

The antibodies used were as follows: anti-SRRM2 mouse monoclonal antibody (S4045) (Sigma-Aldrich, St. Louis, MO) (supplied as SC35 but was recently found to recognize SRRM2 [78] and thus denoted as SRRM2), anti-ATM rabbit monoclonal antibody (2873) (Cell Signaling Technology, Danvers, MA), anti-phospho-ATM mouse monoclonal antibody (Ser1981) (4526) (Cell Signaling Technology), anti-Chk2 mouse monoclonal antibody (3440) (Cell Signaling Technology), anti-phospho-Chk2 rabbit monoclonal antibody (2661) (Cell Signaling Technology), anti-actin rabbit polyclonal antibody (A2066) (Sigma-Aldrich), anti-coilin mouse monoclonal antibody (C1862) (Sigma-Aldrich), anti-PSP1 rabbit monoclonal antibody (supplied by Dr. T. Hirose), anti-γH2AX mouse monoclonal antibody (Merck Millipore, Darmstadt, Germany), anti-DDX21 mouse monoclonal antibody (sc-376,953) (Santa Cruz Biotechnology, Dallas, TX), anti-nucleolin mouse monoclonal antibody (supplied by Dr. S. Yoshimura), and anti-TCOF1 mouse monoclonal antibody (sc-374,536) (Santa Cruz Biotechnology).

### Inhibitors

The Screening Committee of Anticancer Drugs (SCADS) inhibitor kits I–IV (kit I v.3.2, kit II v.2.0, kit III v.1.5, kit IV v.2.3) were provided by the Ministry of Education, Culture, Sports, Science and Technology, Japan. Other inhibitors were obtained as follows: antimycin A (2247–10) (BioVision, Brugg, Switzerland), brequinar (B5707) (Tokyo Chemical Industry, Tokyo, Japan), atovaquone (AK544285) (Ark Pharm, Inc, Libertyville, IL, USA), teriflunomide (163,451–81-8) (Tokyo Chemical Industry), methotrexate (139–13,571) (FUJIFILM Wako Pure Chemical Corporation), cp466722 (25,417) (Cayman Chemical, MI, USA), etoposide (055–08431) (Wako), ve822 (24,198) (Cayman Chemical), rotenone (AK115691) (Ark Pharm, Inc), and NaN_3_ (195–11,092) (FUJIFILM Wako Pure Chemical Corporation).

### siRNA

Cells were transfected with DsiRNAs using Lipofectamine 2000 (Invitrogen, Carlsbad, CA) following the manufacturer’s instructions. DsiRNA against TCOF1 was obtained by Integrated DNA Technologies(5′- rGrGrArCrUrUrGrCrCrArUrCrArArGrCrArUrGrArArArGAA-3′, 5′-rUrUrCrUrUrUrCrArUrGrCrUrUrGrArUrGrGrCrArArGrUrCrCrGrC-3′).

### RNA–fluorescence in situ hybridization (RNA-FISH)

Cells (4 × 10^4^ cells/mL) on coverslips in a 12-well plate were cultured for 24 h following inoculation. The cells were cultured with inhibitors for the indicated time points, fixed with 10% formaldehyde in phosphate-buffered saline (PBS) for 20 min, and permeabilized with 0.1% Triton X-100 in PBS for 10 min. They were washed three times with PBS for 10 min to remove the detergent, once with 2× standard sodium citrate (SSC) for 5 min to replace the buffer content, prehybridized with ULTRAhyb-Oligo Hybridization Buffer (Ambion, Austin, TX) for 1 h at 42°C in a humidified chamber, and incubated overnight with 10 pmol Alexa Fluor 594-labelled oligo-dT_45_ probe (Molecular Probes, Eugene, OR) and diluted with hybridization buffer. Cells were washed for 20 min each at 42°C with 2× SSC, 0.5× SSC, and 0.1× SSC. The nuclei were visualized with 4′, 6-diamidino-2-phenylindole (DAPI). Fluorescent images were captured at random using a Zeiss Axioplan 2 (Carl Zeiss, Jena, Germany) equipped with an OLYMPUS DP70 camera (OLYMPUS, Tokyo, Japan). Confocal microscopy images were acquired using a FLUOVIEW FV10i microscope (OLYMPUS). The ratio of nuclear to total poly(A)^+^ RNA signals was calculated using ImageJ (https://imagej.nih.gov/ij/).

### Immunofluorescence staining

Cells (4 × 10^4^ cells/mL) on coverslips in a 12-well plate were cultured for 24 h following inoculation. They were cultured with inhibitors for the indicated time points, fixed with 10% formaldehyde in PBS for 20 min, and permeabilized in 0.1% Triton X-100 in PBS for 10 min. The cells were washed three times with PBS for 10 min to remove the detergent. Then, the cells were blocked with 6% bovine serum albumin (BSA) in PBS for 1 h at room temperature. Next, coverslips were incubated with the primary antibody in PBS containing 2% BSA, followed by secondary antibodies labelled with Alexa Fluor 488. After secondary antibody incubation, the cells were fixed with 4% formaldehyde in PBS for 10 min. Then, the cells were washed three times with PBS for 10 min to remove the detergent, once with 2× SSC for 5 min to replace the buffer content, prehybridized with ULTRAhyb-Oligo Hybridization Buffer for 1 h at 42°C in a humidified chamber, and incubated overnight with 10 pmol Alexa Fluor 594-labelled oligo-dT_45_ diluted with the hybridization buffer. Cells were washed with 2× SSC, 0.5× SSC, and 0.1× SSC for 5 min each at 42°C. Lastly, nuclei were visualized with DAPI.

### Synthesis of lncRNA probe

The RNA-FISH probes for MALAT1 were synthesized using T3 RNA polymerase and a DIG/FITC RNA labelling kit (Roche Diagnostic, Grenzacherstrasse, Switzerland) according to the manufacturer’s instructions. The RNA-FISH probes for NEAT1 were synthesized by using SP6 RNA polymerase and a DIG/FITC RNA labelling kit. Linearized plasmids (1 μg) containing MALAT1 fragment (1075–4780 nts in NR_002819.4) or NEAT1 fragment (1–1000 nts in EF177379) were used as templates for the transcription of MALAT1 or NEAT1 RNA probe, respectively. The MALAT1 plasmid was obtained from Dr. N. Akimitsu, and the NEAT1 plasmid was obtained from Dr. T. Hirose.

### Immunofluorescence staining of lncRNA

Cells (4 × 10^4^ cells/mL) on coverslips in a 12-well plate were cultured for 24 h following inoculation. The cells were cultured with inhibitor for 36 h, fixed with 10% formaldehyde in PBS for 20 min, and permeabilized in 0.1% Triton X-100 in PBS for 10 min. Cells were prehybridized with ULTRAhyb-Oligo Hybridization Buffer for 1 h at 55°C in a humidified chamber and then incubated overnight with 2.8 µg/mL lncRNA probe diluted with hybridization buffer. Then, cells were washed two times with 50% formamide/SSC with 0.1% tween 20 for 30 min at 55°C, two times with NTET buffer (10-mM Tris-HCl, 1-mM EDTA, 0.5-M NaCl, 0.1% tween 20) for 15 min at 37°C, once with 2× SSC with 0.1% tween 20 for 30 min at 55°C, once with 0.1× SSC with 0.1% tween 20 for 30 min at 55°C, and once with PBS at room temperature. The cells were blocked with 6% BSA in PBS for 1 h at room temperature. The coverslips were incubated with the anti-DIG1 antibody in PBS containing 2% BSA, followed by secondary antibodies labelled with Alexa Fluor 594. After secondary antibodies incubation, cells were fixed with 4% formaldehyde in PBS for 10 min. Cells were washed once with PBS for 10 min, twice with 2 × SSC for 5 min to replace the buffer content, prehybridized with ULTRAhyb-Oligo Hybridization Buffer for 1 h at 42°C in a humidified chamber, and then incubated overnight with 10 pmol Alexa Fluor 488-labelled oligo-dT45 diluted with hybridization buffer. After incubation, the cells were washed for 5 min each at 42°C with 2× SSC, 0.5× SSC, and 0.1× SSC. Eventually, the nuclei were visualized with DAPI.

### Western blotting

Cultured cells were collected, washed with PBS, and sonicated using a Microson Ultrasonic Cell Disruptor (Misonix). Whole-cell extracts were separated by SDS-PAGE and electrotransferred to FluoroTrans Polyvinylidene Fluoride or Polyvinylidene Difluoride (PVDF) Transfer Membranes (Pall, Ann Arbor, MI) using a BioRad Trans-Blot (BioRad, Hercules, CA). The blotted PVDF membrane was blocked with 5% foetal bovine serum/tris-buffered saline (TBS) containing 0.1% Tween 20 for 1 h at room temperature and reacted with the primary antibody with continuous rotation overnight at 4°C. The blotted membrane was washed three times with TBS containing 0.1% Tween 20 for 10 min each and incubated with horseradish peroxidase-conjugated secondary antibody for 3 h at room temperature with continuous rotation. The blotted membrane was washed three times with TBS containing 0.1% Tween 20 for 10 min each, reacted with chemiluminescence reagent (Millipore, Darmstadt, Germany), and detected using an image analyser LAS 4000 mini (GE Healthcare, Chicago, IL).

### DHODH assay

DHODH activity was measured using a modified protocol according to Yin et al. [34]. Briefly, cells were collected, washed with PBS, resuspended in extraction buffer (0.25-M sucrose, 20-mM HEPES (4-(2-hydroxyethyl)-1-piperazineethanesulfonic acid)–KOH, pH 7.5, 10-mM KCl, 1.5-mM MgCl_2_, 1-mM ethylenediaminetetraacetic acid (EDTA), 1-mM, dithiothreitol and 0.1-mM phenylmethylsulfonyl fluoride (PMSF)) and homogenized using a Teflon-glass homogenizer. The homogenate was centrifuged twice at 800 × g for 10 min, and the supernatant was centrifuged at 10,000 × g for 15 min. The pellet was resuspended in the extraction buffer and pre-incubated with an inhibitor for 2 h at 37°C. Then, 160-mM K_2_CO_3_/HCl, pH 8.0, 40-μM dihydroorotate, and 80-μM decylubiquinone were added for 1 h at 37°C. The reference sample was kept on ice. The reaction mixture was supplemented with 10-mM K_2_CO_3_, 2-mM K_3_[Fe(CN)_6_], and 1-mM 4-(trifluoromethyl) benzamidoxime (4-TFMBAO) and heated at 80°C for 4 min. The reaction was stopped by cooling on ice, and the fluorescence intensity was measured using SYNERGY H1MF (Biotek, CA, USA); excitation and emission wavelengths were 340 and 460 nm, respectively.

## Supplementary Material

Supplemental Material

## Data Availability

Data supporting the findings of this study are available within the article.

## References

[1] Orphanides G, Reinberg D. A unified theory of gene expression cell. Cell. 2002;108(4):439–451. 11909516 10.1016/s0092-8674(02)00655-4

[2] Millevoi S, Vagner S. Molecular mechanisms of eukaryotic pre-mRNA 3’ end processing regulation. Nucleic Acids Res. 2009;38(9):2757–2774. 20044349 10.1093/nar/gkp1176 PMC2874999

[3] Bentley DL. Coupling mRNA processing with transcription in time and space. Nat Rev Genet. 2014;15(3):163–175. 24514444 10.1038/nrg3662 PMC4304646

[4] Kaida D, Motoyoshi H, Tashiro E, et al. Spliceostatin A targets SF3b and inhibits both splicing and nuclear retention of pre-mRNA Nat. Chem Biol. 2007;3:576–583. 10.1038/nchembio.2007.18 17643111

[5] Kotake Y, Sagane K, Owa T, et al. Splicing factor SF3b as a target of the antitumor natural product pladienolide. Nat Chem Biol. 2007;3(9):570–575. 17643112 10.1038/nchembio.2007.16

[6] Hasegawa M, Miura T, Kuzuya K, et al. Identification of SAP155 as the Target of GEX1A (Herboxidienean) Antitumor Natural Product ACS Chem. Biol. 2011;6:229–233. 10.1021/cb100248e 21138297

[7] Seiler M, Yoshimi A, Darman R, et al. H3B-8800, an orally available small-molecule splicing modulator, induces lethality in spliceosome-mutant cancers Nat. Med. 2018;24:497–504. 10.1038/nm.4493 PMC6730556 29457796

[8] Kahles A, Lehmann KV, Toussaint NC, et al. Comprehensive analysis of alternative splicing across tumors from 8,705 patients. Cancer Cell. 2018;34(2):211–224. 30078747 10.1016/j.ccell.2018.07.001 PMC9844097

[9] Xin P, Xu X, Deng C, et al. The role of JAK/STAT signaling pathway and its inhibitors in diseases. Int Immunopharmacol. 2020;80:106210. 31972425 10.1016/j.intimp.2020.106210

[10] Fujiwara N, Yoshikawa M, Yamazaki T, et al. A screening method tuned for mrna processing factors in human cells by evaluation of the luciferase reporter activity and the subcellular distribution of bulk poly(A) + RNA. Biotchnol Biochem. 2010;74(7):1512–1516. 10.1271/bbb.100363 20622428

[11] Fujita KI, Okamura M, Nishimoto S, et al. Establishment of a Monitoring System to Detect Inhibition of mRNA Processing Biosci. Biotechnol Biochem. 2012;76(6):1248–1251. 10.1271/bbb.120226 22790958

[12] Kurata M, Fujiwara N, Fujita KI, et al. Food-derived compounds apigenin and luteolin modulate mrna splicing of introns with weak splice sites.iScience. 2019;22:336–352. 31809999 10.1016/j.isci.2019.11.033 PMC6909097

[13] Evans RD, Guy HI. Mammalian pyrimidine biosynthesis: fresh insights into an ancient pathway. J Biol Chem. 2004;279(32):33035–33038. 15096496 10.1074/jbc.R400007200

[14] Alcázar-Fabra M, Navas P, Brea-Calvo G. Coenzyme Q biosynthesis and its role in the respiratory chain structure.Biochimica Biophys Acta Bioenerg 2016;1857(8):1073–1078 10.1016/j.bbabio.2016.03.010 26970214

[15] Singh A, Maqbool M, Mobashir M, et al. Dihydroorotate dehydrogenase: a drug target for the development of antimalarials. Eur J Med Chem. 2017;125:640–651. 27721149 10.1016/j.ejmech.2016.09.085

[16] Lolli LM, Sainas S, Pippione CA, et al. Use of human dihydroorotate dehydrogenase (hdhodh) inhibitors in autoimmune diseases and new perspectives in cancer therapy recent pat. Anticancer Drug Discov. 2018;13:86–105. 10.2174/1574892812666171108124218 29119937

[17] Mao C, Liu X, Zhang Y, et al. DHODH-mediated ferroptosis defence is a targetable vulnerability in cancer. Nature. 2021;593(7860):586–590. 33981038 10.1038/s41586-021-03539-7 PMC8895686

[18] Sykes BD, Kfoury SY, Mercier EF, et al. Inhibition of dihydroorotate dehydrogenase overcomes differentiation blockade in acute myeloid leukemia. cell. 2016;167:171–186. 27641501 10.1016/j.cell.2016.08.057 PMC7360335

[19] Yang CF, Gopula B, Liang JJ, et al. Novel AR-12 derivatives, P12-23 and P12-34, inhibit flavivirus replication by blocking host de novo pyrimidine biosynthesis. Emerg Microbes Infect. 2018;7(1):187. 30459406 10.1038/s41426-018-0191-1 PMC6246607

[20] Luthra P, Naidoo J, Pietzsch AC, et al. Inhibiting pyrimidine biosynthesis impairs Ebola virus replication through depletion of nucleoside pools and activation of innate immune responses.Antiviral Res. 2018; 158: 288–302. 30144461 10.1016/j.antiviral.2018.08.012 PMC6436837

[21] Knight AD, Hejmanowski QA, Dierksheide EJ, et al. Inhibition of herpes simplex virus type 1 by the experimental immunosuppressive agent leflunomide Transplantation. 2001;71:170–174. 10.1097/00007890-200101150-00031 11211189

[22] Fu H, Zhang Z, Dai Y, et al. Brequinar inhibits enterovirus replication by targeting biosynthesis pathway of pyrimidines. Am J Transl Res. 2020;12(12):8247–8255. 33437396 PMC7791496

[23] Park JG, Ávila-Pérez G, Nogales A, et al. Identification and characterization of novel compounds with broad-spectrum antiviral activity against influenza A and. B Viruses J Virol. 2020;94(7):e02149–19. 31941776 10.1128/JVI.02149-19 PMC7081893

[24] Luban J, Sattler AR, Mühlberger E. The DHODH inhibitor PTC299 arrests SARS-CoV-2 replication and suppresses induction of inflammatory cytokines. Virus Res. 2021;292:198246. 33249060 10.1016/j.virusres.2020.198246 PMC7690341

[25] Xiong R, Zhang L, Li S. Novel and potent inhibitors targeting DHODH are broad-spectrum antivirals against RNA viruses including newly-emerged coronavirus SARS-CoV-2. Protein Cell. 2020;11(10):723–739. 32754890 10.1007/s13238-020-00768-w PMC7402641

[26] Johnson C, Primorac D, McKinstry M, et al. Tracking COL1A1 RNA in osteogenesis imperfecta. splice-defective transcripts initiate transport from the gene but are retained within the SC35 domain. J Cell Biol. 2000;150(3):417–432. 10931857 10.1083/jcb.150.3.417 PMC2175183

[27] Zhang X, Hamblin MH, Yin KJ. The long noncoding RNA Malat1: its physiological and pathophysiological functions. RNA Biol. 2017;14(12):1705–1714. 28837398 10.1080/15476286.2017.1358347 PMC5731810

[28] Hirose T, Yamazaki T, Nakagawa S. Molecular anatomy of the architectural NEAT1 noncoding RNA: the domains, interactors, and biogenesis pathway required to build phase-separated nuclear paraspeckles. Wiley Interdiscip Rev RNA. 2019;10(6):e1545. 31044562 10.1002/wrna.1545

[29] Andrew SG, Phi MD, Zunamys IC, et al. Coilin participates in the suppression of RNA polymerase I in response to cisplatin-induced DNA damage. Mol Biol Cell. 2011;22(7):1070–1079. 21289084 10.1091/mbc.E10-08-0731 PMC3069010

[30] Martin J, Ajit SD, Shona M, et al. Mitochondrial proton and electron leaks. Essays Biochem. 2010;47:53–67. 20533900 10.1042/bse0470053 PMC3122475

[31] Lydia GH, John F. Flaherty Atovaquone: a review . Ann Pharmacother. 1993;27(12):1488–1494 8305784 10.1177/106002809302701215

[32] Anastasia AK, Vladimir VR, Boris VC, et al. Pyrimidine biosynthesis links mitochondrial respiration to the p53 pathway. Proc Natl Acad Sci U S A. 2010;107(29):12828–12833. 20566882 10.1073/pnas.0910885107 PMC2919937

[33] Bruneau JM, Yea CM, Spinella-Jaegle S, et al. Purification of human dihydro-orotate dehydrogenase and its inhibition by A77 1726, the active metabolite of leflunomide. Biochem J. 1998;336(2):299–303. 9820804 10.1042/bj3360299 PMC1219871

[34] Sheng Y, Tsutomu K, Qinchang Z, et al. Fluorescence assay of dihydroorotate dehydrogenase that may become a cancer biomarker. Sci Rep. 2017;7:40670. 28084471 10.1038/srep40670 PMC5233952

[35] Henghe T, Bruce NC. Understanding the mechanisms of action of methotrexate implications for the treatment of rheumatoid arthritis. Bull Hosp Jt Dis. 2007;65(3):168–173. 17922664

[36] Deans MR, Morgens WD, Ökesli A, et al. Parallel shRNA and CRISPR-Cas9 screens enable antiviral drug target identification. Nat Chem Biol. 2016;12(5):361–366. 27018887 10.1038/nchembio.2050 PMC4836973

[37] Arnould S, Rodier G, Matar G, et al. Checkpoint kinase 1 inhibition sensitises transformed cells to dihydroorotate dehydrogenase inhibition. Oncotarget. 2017;8(56):95206–95222. 29221122 10.18632/oncotarget.19199 PMC5707016

[38] Lafita-Navarro MC, Venkateswaran N, Kilgore JA, et al. Inhibition of the de novo pyrimidine biosynthesis pathway limits ribosomal RNA transcription causing nucleolar stress in glioblastoma cells. PLoS Genet. 2020;16(11):e1009117. 33201894 10.1371/journal.pgen.1009117 PMC7707548

[39] Hubackova S, Davidova E, Boukalova S, et al. Replication and ribosomal stress induced by targeting pyrimidine synthesis and cellular checkpoints suppress p53-deficient tumors. Cell Death Dis. 2020;11(2):110. 32034120 10.1038/s41419-020-2224-7 PMC7007433

[40] Berger DN, Stanley FKT, Moore S, et al. ATM-dependent pathways of chromatin remodelling and oxidative DNA damage responses. Philos Trans R Soc Lond B Biol Sci. 2017;372(1731):20160283. 28847820 10.1098/rstb.2016.0283 PMC5577461

[41] Ladds MJGW, van Leeuwen IMM, Drummond CJ, et al. A DHODH inhibitor increases p53 synthesis and enhances tumor cell killing by p53 degradation blockage. Nat Commun. 2018;9(1):1107. 29549331 10.1038/s41467-018-03441-3 PMC5856786

[42] Rainey MD, Charlton ME, Stanton RV, et al. Transient inhibition of ATM kinase is sufficient to enhance cellular sensitivity to ionizing radiation. Cancer Res. 2008;68(18):7466–7474. 18794134 10.1158/0008-5472.CAN-08-0763 PMC2559948

[43] Zhang A, Lyu YL, Lin CP, et al. A protease pathway for the repair of topoisomerase II-DNA covalent complexes. J Biol Chem. 2006;281(47):35997–36003. 16973621 10.1074/jbc.M604149200

[44] Tanaka T, Halicka HD, Traganos F, et al. Induction of ATM activation, histone H2AX phosphorylation and apoptosis by etoposide: relation to cell cycle phase. Cell cycle. 2007;6(3):371–376. 17297310 10.4161/cc.6.3.3835

[45] Kurz EU, Lees-Miller SP. DNA damage-induced activation of ATM and ATM-dependent signaling pathways.DNA Repair (Amst). 2004;3(8–9):889–900. 15279774 10.1016/j.dnarep.2004.03.029

[46] Fokas E, Prevo R, Pollard JR, et al. Targeting ATR in vivo using the novel inhibitor VE-822 results in selective sensitization of pancreatic tumors to radiation. Cell Death Dis. 2012;3(12):e441. 23222511 10.1038/cddis.2012.181 PMC3542617

[47] Sharma A, Singh K, Almasan A. Histone H2AX phosphorylation: a marker for DNA damage Methods. Mol Biol. 2012;920:613–626. 10.1007/978-1-61779-998-3_40 22941631

[48] Santoriello C, Sporrij A, Yang S, et al. RNA helicase DDX21 mediates nucleotide stress responses in neural crest and melanoma cells. Nat Cell Biol. 2020;22(4):372–379. 32231306 10.1038/s41556-020-0493-0 PMC7185069

[49] Jia W, Yao Z, Zhao J, et al. New perspectives of physiological and pathological functions of nucleolin (NCL. Life Sci. 2017;186:1–10. 28751161 10.1016/j.lfs.2017.07.025

[50] Grzanka M, Piekiełko-Witkowska A. The Role of TCOF1 Gene in Health and Disease. Int J Mol Sci. 2021;22(5):2482. 33804586 10.3390/ijms22052482 PMC7957619

[51] Korsholm LM, Gál Z, Lin L, et al. Double-strand breaks in ribosomal RNA genes activate a distinct signaling and chromatin response to facilitate nucleolar restructuring and repair. Nucleic Acids Res. 2019 Sep 5 47(15):8019–8035. 31184714 10.1093/nar/gkz518 PMC6735822

[52] Calo E, Gu B, Bowen ME, et al. Tissue-selective effects of nucleolar stress and rDNA damage in developmental disorders. Nature. 2018;554(7690):112–117. 29364875 10.1038/nature25449 PMC5927778

[53] Inoue A, Tsugawa K, Tokunaga K, et al. S1-1 nuclear domains: characterization and dynamics as a function of transcriptional activity. Biol Cell. 2008;100(9):523–535. 18315527 10.1042/BC20070142

[54] Hsiang YH, Hertzberg R, Hecht S, et al. Camptothecin induces protein-linked DNA breaks via mammalian DNA topoisomerase I. J Biol Chem. 1985;260(27):14873–14878. 2997227

[55] Diouf B, Lin W, Goktug A, et al. Alteration of RNA splicing by small-molecule inhibitors of the interaction between NHP2L1 and U4. SLAS Discov. 2018;23(2):164–173. 28985478 10.1177/2472555217735035 PMC5783296

[56] Francis LK, Alsayed Y, Leleu X, et al. Combination mammalian target of rapamycin inhibitor rapamycin and HSP90 inhibitor 17-allylamino-17-demethoxygeldanamycin has synergistic activity in multiple myeloma. Clin Cancer Res. 2006;12(22):6826–6835. 17121904 10.1158/1078-0432.CCR-06-1331

[57] Ferraldeschi R, Welti J, Powers MV, et al. Second-generation hsp90 inhibitor onalespib blocks mrna splicing of androgen receptor variant 7 in prostate cancer cells. Cancer Res. 2016;76(9):2731–2742. 27197266 10.1158/0008-5472.CAN-15-2186 PMC4874658

[58] Lindell TJ, Weinberg F, Morris PW, et al. Specific inhibition of nuclear RNA polymerase II by alpha-amanitin. Science. 1970;170(3956):447–449. 4918258 10.1126/science.170.3956.447

[59] Carmo-Fonseca M, Pepperkok R, Carvalho MT, et al. Transcription-dependent colocalization of the U1, U2, U4/U6, and U5 snRNPs in coiled bodies. J Cell Biol. 1992;117(1):1–14. 1532583 10.1083/jcb.117.1.1 PMC2289407

[60] Wang P, Lou PJ, Leu S, et al. Modulation of alternative pre-mRNA splicing in vivo by pinin. Biochem Biophys Res Commun. 2002;294(2):448–455. 12051732 10.1016/S0006-291X(02)00495-3

[61] Spector DL. Nuclear organization and gene expression. Exp Cell Res. 1996;229(2):189–197. 8986596 10.1006/excr.1996.0358

[62] Dabo S, Meurs EF. dsRNA-dependent protein kinase PKR and its role in stress, signaling and HCV infection. Viruses. 2012;4(11):2598–2635. 23202496 10.3390/v4112598 PMC3509664

[63] Ilan L, Osman F, Namer LS, et al. PKR activation and eIF2α phosphorylation mediate human globin mRNA splicing at spliceosome assembly. Cell Res. 2017;27(5):688–704. 28374749 10.1038/cr.2017.39 PMC5520854

[64] Murphy T, Yee KWL. Cytarabine and daunorubicin for the treatment of acute myeloid leukemia. Expert Opin Pharmacother. 2017;18(16):1765–1780. 29017371 10.1080/14656566.2017.1391216

[65] Wander DPA, van der Zanden SY, van der Marel GA, et al. Doxorubicin and Aclarubicin: shuffling Anthracycline Glycans for Improved Anticancer Agents. J Med Chem. 2020;63(21):12814–12829. 33064004 10.1021/acs.jmedchem.0c01191 PMC7667640

[66] van der Zanden Sy, Qiao X, Neefjes J. New insights into the activities and toxicities of the old anticancer drug doxorubicin. FEBS J. 2021;288(21):6095–6111. 33022843 10.1111/febs.15583 PMC8597086

[67] Lee JS, Lin YY, Wang TS, et al. Antitumorigenic Effects of ZAKβ, an Alternative Splicing Isoform of ZAK. Chin J Physiol. 2018;61(1):25–34. 29374956 10.4077/CJP.2018.BAG528

[68] Jiang D, Lynch C, Medeiros BC, et al. Identification of doxorubicin as an inhibitor of the IRE1α-XBP1 axis of the unfolded protein response. Sci Rep. 2016;6(1):33353. 27634301 10.1038/srep33353 PMC5025885

[69] Chatham JC, Young ME, Zhang J. Role of O-linked N-acetylglucosamine (O-GlcNAc) modification of proteins in diabetic cardiovascular complications. Curr Opin Pharmacol. 2021;57:1–12. 32937226 10.1016/j.coph.2020.08.005 PMC9027139

[70] Bennett EP, Mandel U, Clausen H, et al. Control of mucin-type O-glycosylation: a classification of the polypeptide GalNAc-transferase gene family. Glycobiology. 2012;22(6):736–756. 22183981 10.1093/glycob/cwr182 PMC3409716

[71] Chen J, Yang S. Catalytic mechanism of UDP-glucose dehydrogenase. Biochem Soc Trans. 2019;47(3):945–955. 31189734 10.1042/BST20190257

[72] Lewis BA, Burlingame AL, Myers SA. Human RNA Polymerase II Promoter Recruitment in Vitro Is Regulated by O-Linked N-Acetylglucosaminyltransferase (OGT). J Biol Chem. 2016;291(27):14056–14061. 27129214 10.1074/jbc.M115.684365 PMC4933165

[73] Echizenya S, Ishii Y, Kitazawa S, et al. Discovery of a new pyrimidine synthesis inhibitor eradicating glioblastoma-initiating cells. Neuro Oncol. 2020;22(2):229–239. 31499527 10.1093/neuonc/noz170 PMC7442393

[74] Madak JT, Bankhead A 3rd, Cuthbertson CR, et al. Revisiting the role of dihydroorotate dehydrogenase as a therapeutic target for cancer. Pharmacol Ther. 2019;195:111–131. 30347213 10.1016/j.pharmthera.2018.10.012

[75] Zhou Y, Tao L, Zhou X, et al. DHODH and cancer: promising prospects to be explored. Cancer Metab. 2021;9(1):22. 33971967 10.1186/s40170-021-00250-z PMC8107416

[76] Bar-Or A, Pachner A, Menguy-Vacheron F, et al. Teriflunomide and its mechanism of action in multiple sclerosis. Drugs. 2014;74(6):659–674. 24740824 10.1007/s40265-014-0212-x PMC4003395

[77] Alamri RD, Elmeligy MA, Albalawi GA, et al. Leflunomide an immunomodulator with antineoplastic and antiviral potentials but drug-induced liver injury: a comprehensive review. Int Immunopharmacol. 2021;93:107398. 33571819 10.1016/j.intimp.2021.107398 PMC7869628

[78] Ilik İA, Malszycki M, Lübke AK, et al. SON and SRRM2 are essential for nuclear speckle formation. Elife. 2020;9:e60579. 33095160 10.7554/eLife.60579 PMC7671692

