# Supplementary material for: Inhibition of mitochondrial complex III or dihydroorotate dehydrogenase (DHODH) triggers formation of poly(A)+ RNA foci adjacent to nuclear speckles following activation of ATM (ataxia telangiectasia mutated)
Source: RNA Biol. 2022 Nov 22;19(1):1244–55. doi: 10.1080/15476286.2022.2146919 (PMC9683070; doi:10.1080/15476286.2022.2146919)
Supplement: Supplemental Material [file KRNB_A_2146919_SM2131.zip › Rerevised_Supplemental_Figure_Legend.docx]

**Supplemental Figure Legends**

Supplemental Figure 1. Nuclear localization of bulk poly(A)^+^ RNA treated with antimycin A

(A), The localization analysis of bulk poly(A)^+^ RNA with MALAT1. Poly(A)^+^ RNA (green), MALAT1 (red) and chromosomal DNA (blue) were visualized in U2OS cells. Cells were treated with DMSO, antimycin A (0.25 µM) for 36 h. Scale bar, 10 μm. In the right panels, signal intensities were plotted with white lines in the left panels. Signal intensities on the white line for poly(A)^+^ RNA and MALAT1 were plotted and presented with red and green lines in the right panels, respectively. (B), The localization analysis of bulk poly(A)^+^ RNA with PSP1. Poly(A)^+^ RNA (red), PSP1 (green), and chromosomal DNA (blue) were visualized in U2OS cells. Cells were treated with DMSO or antimycin A (0.25 µM) for 24 h. Scale bar, 10 μm. In the right panels, signal intensities were plotted with white lines in the left panels. Signal intensities on the white line for poly(A)^+^ RNA and PSP1 were plotted and presented with red and green lines in the right panels, respectively. (C), The localization analysis of bulk poly(A)^+^ RNA with NEAT1. Poly(A)^+^ RNA (green), NEAT1 (red), and chromosomal DNA (blue) were visualized in U2OS cells. Cells were treated with DMSO, antimycin A (0.25 µM) for 36 h. Scale bar, 10 μm. In the right panels, signal intensities were plotted with white lines in the left panels. Signal intensities on the white line for poly(A)^+^ RNA and NEAT1 were plotted and presented with red and green lines in the right panels, respectively. (D), The localization analysis of bulk poly(A)^+^ RNA with coilin. Poly(A)^+^ RNA (red), coilin (green), and chromosomal DNA (blue) were visualized in U2OS cells. Cells were treated with DMSO or antimycin A (0.25 µM) for 24 h. Scale bar, 10 μm. In the right panels, signal intensities were plotted with white lines in the left panels. Signal intensities on the white line for poly(A)^+^ RNA and coilin were plotted and presented with red and green lines in the right panels, respectively.

Supplemental Figure 2. Antimycin induces nuclear poly(A)^+^ RNA accumulation in a variety of cells

(A) - (C), The concentration- and time-dependent accumulation of nuclear poly(A)^+^ RNA in U2OS (A), HeLa (B), and MCF7 (C) were analyzed. The cells were treated with DMSO or antimycin A for 12, 24, or 36 h. Signal intensities of the whole cell and the nucleus were quantified using ImageJ (n = 40). Boxes show median (centerline) as well as upper and lower quartiles. Whiskers show the lowest and highest values. Statistical analysis was performed using one-way ANOVA followed by Dunnett’s test. ****p* < 0.001

Supplemental Figure 3. DHODH inhibitor induced nuclear poly(A)^+^ RNA accumulation adjacent to the speckle.

(A) RNA-FISH was performed to determine the localization of bulk poly(A)^+^ RNA. MCF7 cells were treated with DMSO, atovaquone (10 µM), teriflunomide (75 µM), or methotrexate (0.25 µM) for 36 h. The bulk poly(A)^+^ RNA was visualized with Alexa Fluor 594-labeled oligo-dT_45_ probe. The nuclei were visualized with DAPI. Scale bar, 50 μm. (B) The ratio of the nuclear distribution of poly(A)^+^ RNA was analyzed. Signal intensities of the whole cell and the nucleus were quantified using ImageJ (n = 40). Boxes show median (centerline) as well as upper and lower quartiles. Whiskers show the lowest and highest values. Statistical analysis was performed using one-way ANOVA followed by Dunnett’s test. ****p* < 0.001. (C) The localization of bulk poly(A)^+^ RNA and nuclear speckles. Poly(A)^+^ RNA (red), speckles (green), and chromosomal DNA (blue) were visualized in MCF7 cells. Cells were treated with DMSO, atovaquone (10 µM), or teriflunomide (75 µM) for 36 h. Scale bar, 10 μm. Signal intensities on the white line for poly(A)^+^ RNA and speckle in the left panels are plotted and presented with red and green lines in the right panels, respectively. (D) The evaluation of DHODH activity in DMSO, antimycin A, brequinar, atovaquone, and teriflunomide-treated cell lysates (n = 3). Statistical analysis was performed using one-way ANOVA followed by Dunnett’s test. ****p* < 0.001. Atova: atovaquone, Teri: teriflunomide, Metho: methotrexate, Anti: antimycin A, Bre: brequinar

Supplemental Figure 4. ATR inhibitor partially attenuates nuclear poly(A)^+^  RNA accumulation

(A) RNA-FISH was performed to determine the localization of bulk poly(A)^+^ RNA. MCF7 cells were treated with DMSO, antimycin A (0.25 µM), or brequinar (0.5 µM), with or without VE822 (50 nM) for 36 h. The bulk poly(A)^+^ RNA was visualized with Alexa Fluor 594-labeled oligo-dT_45_ probe. The nuclei were visualized with DAPI. Scale bar, 50 μm. (B) The ratio of the nuclear distribution of poly(A)^+^ RNA was analyzed. Signal intensities of the whole cell and the nucleus were quantified using ImageJ (n = 40). Boxes show median (centerline) as well as upper and lower quartiles. Whiskers show the lowest and highest values. Statistical analysis was performed using one-way ANOVA followed by Tukey’s test. ****p* < 0.001. Anti: antimycin A, Bre: brequinar

Supplemental Figure 5. Relocalization of DDX21 and nucleolin with the DHODH inhibitors treatment

(A) Localization of bulk poly(A)^+^ RNA and nucleolin. Poly(A)^+^ RNA (red), nucleolin (green), and chromosomal DNA (blue) were visualized in MCF7 cells. Cells were treated with DMSO, antimycin A (0.25 µM), brequinar (0.5 µM), or etoposide (5 µM) with or without　CP466722 (20µM) for 36 h. The bulk poly(A)^+^ RNA was visualized with Alexa Fluor 594-labeled oligo-dT_45_ probe. The nuclei were visualized with DAPI. Scale bar, 50 μm. (B), (C) The localization of bulk poly(A)^+^ RNA and DDX21 (B) or nucleolin (C). Poly(A)^+^ RNA (red), DDX21 (green), nucleolin (green), and chromosomal DNA (blue) were visualized in MCF7 cells. Cells were treated with DMSO, antimycin A (0.25 µM), or brequinar (0.5 µM) for 36 h. Scale bar, 10 μm. Signal intensities on the white line are plotted and presented with red (poly(A)^+^ RNA) and green (DDX21 in B and nucleolin in C) lines in the right panel, respectively.

Supplemental Figure 6. Effect of TCOF1 for poly(A)^+^ RNA accumulation in the nucleus

(A) The localization of bulk poly(A)^+^ RNA and TCOF1. Poly(A)^+^ RNA (red), TCOF1 (green), and chromosomal DNA (blue) were visualized in MCF7 cells. Cells were treated with DMSO or brequinar (0.5 µM) for 36 h. Scale bar, 10 μm. (B) MCF7 cells were treated with DMSO or brequinar (0.5 µM) for 36 h, with or without TCOF1 knockdown. The bulk poly(A)^+^ RNA was visualized with Alexa Fluor 594-labeled oligo-dT_45_ probe. The nuclei were visualized with DAPI. Scale bar, 50 μm. (C) MCF7 cells were treated with DMSO, brequinar (0.5 µM), or actinomycin D (2ng/mL) for 36 h. The bulk poly(A)^+^ RNA was visualized by Alexa Fluor 594-labeled oligo-dT_45_ probe. The nuclei were visualized with DAPI. Scale bar, 50 μm. (D) The ratio of the nuclear distribution of poly(A)^+^ RNA was analyzed. Signal intensities of the whole cell and the nucleus were quantified using ImageJ (n = 40). Boxes show median (centerline) as well as upper and lower quartiles. Whiskers show the lowest and highest values. Statistical analysis was performed using one-way ANOVA followed by Dunnett’s test. ****p* < 0.001. Bre: brequinar, ActD: actinomycin D
