# Supplementary figures and images for: Inhibition of mitochondrial complex III or dihydroorotate dehydrogenase (DHODH) triggers formation of poly(A)+ RNA foci adjacent to nuclear speckles following activation of ATM (ataxia telangiectasia mutated)
Source: RNA Biol. 2022 Nov 22;19(1):1244–55. doi: 10.1080/15476286.2022.2146919 (PMC9683070; doi:10.1080/15476286.2022.2146919)

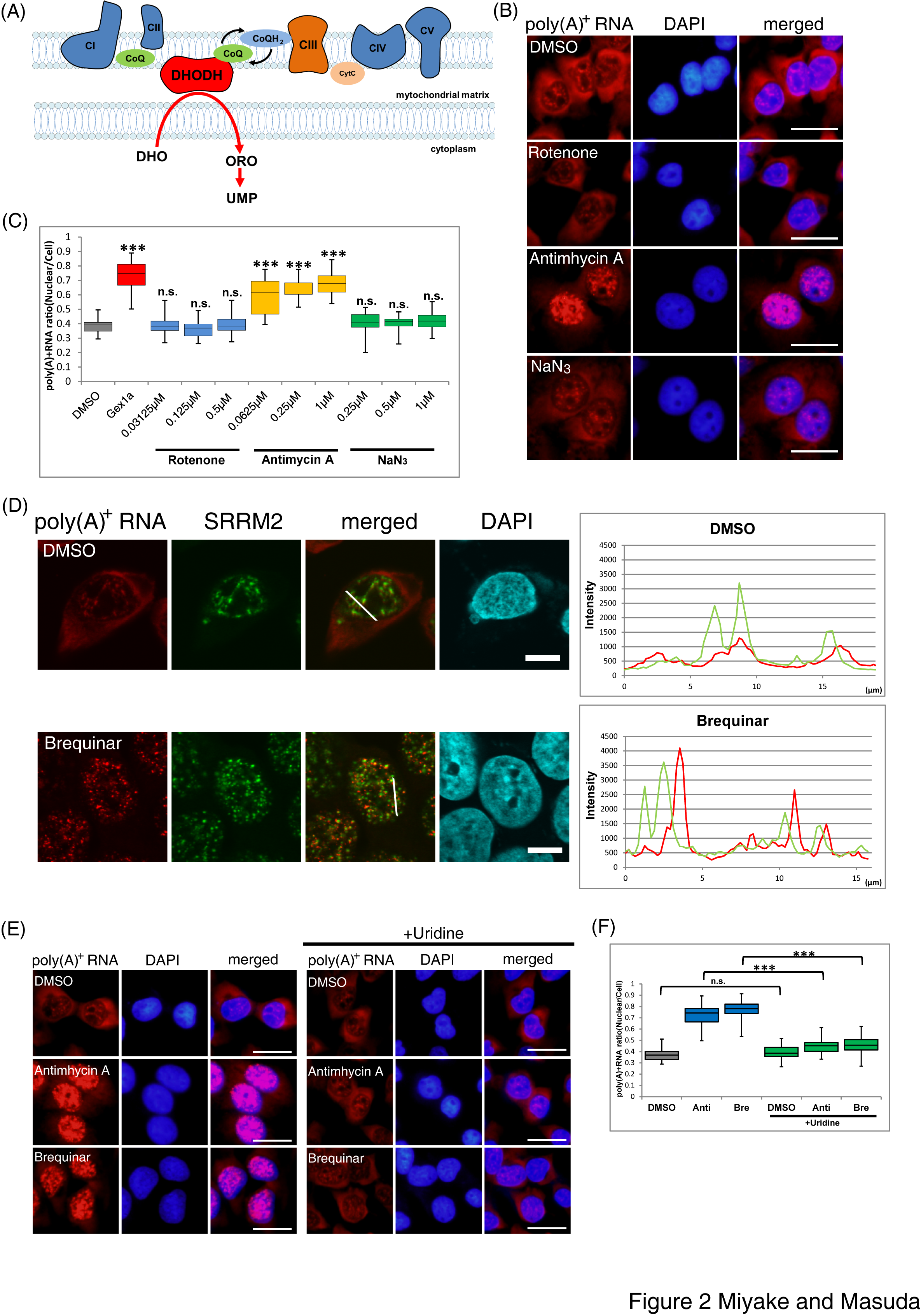

Supplement: Supplemental Material [file KRNB_A_2146919_SM2131.zip › sup_Fig.2.tif]

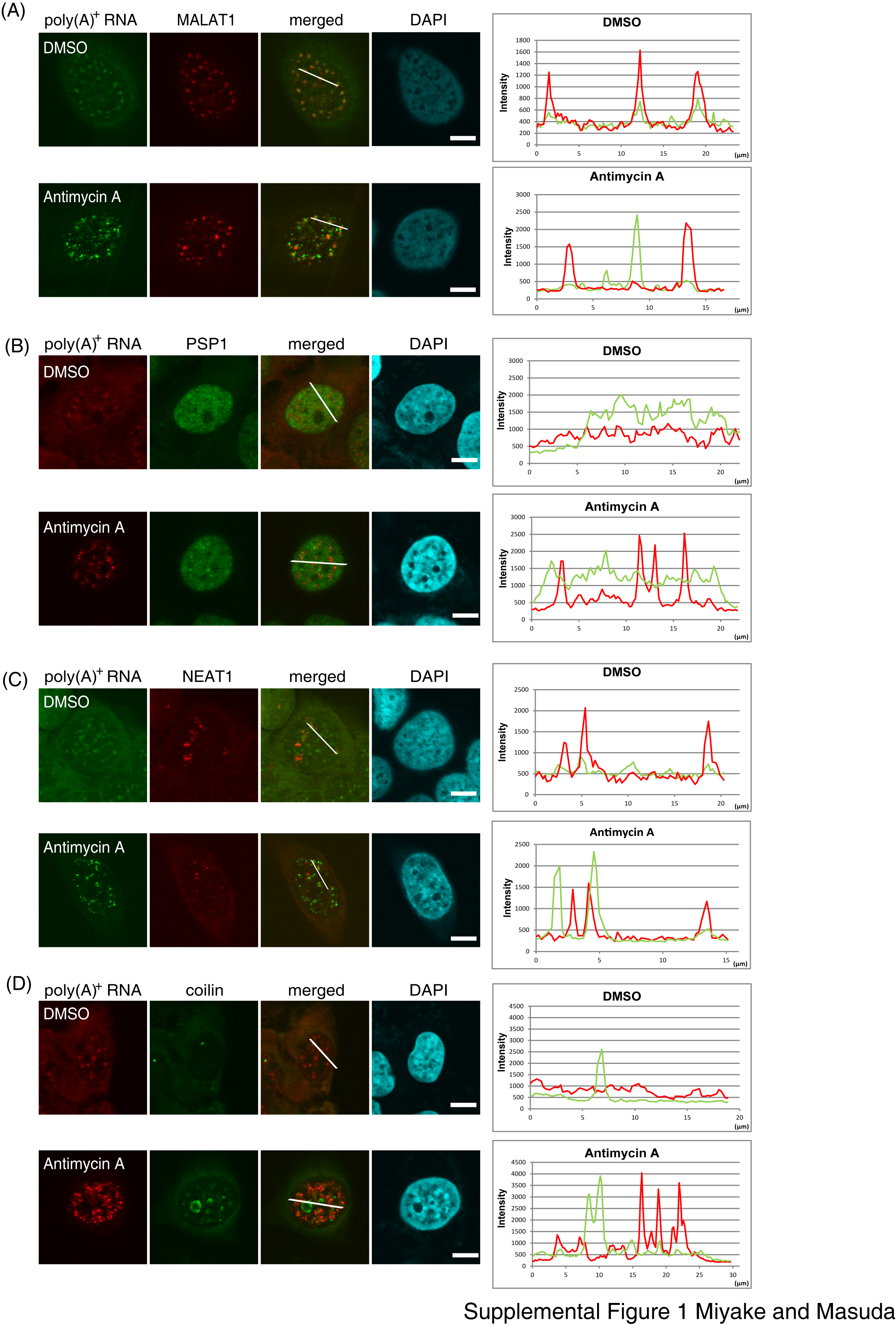

Supplement: Supplemental Material [file KRNB_A_2146919_SM2131.zip › sup-Fig.1.tif]

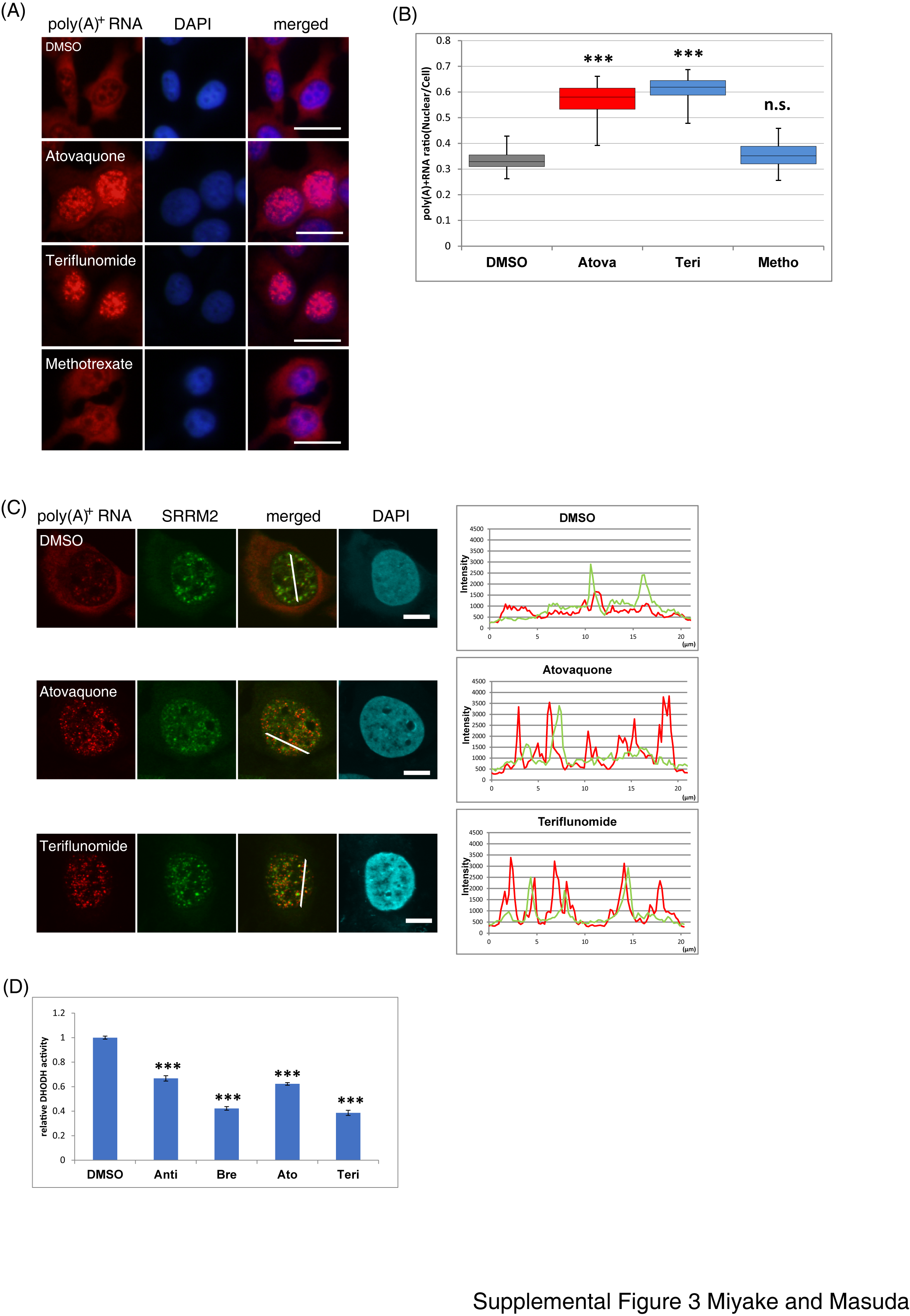

Supplement: Supplemental Material [file KRNB_A_2146919_SM2131.zip › sup-Fig.3.tif]

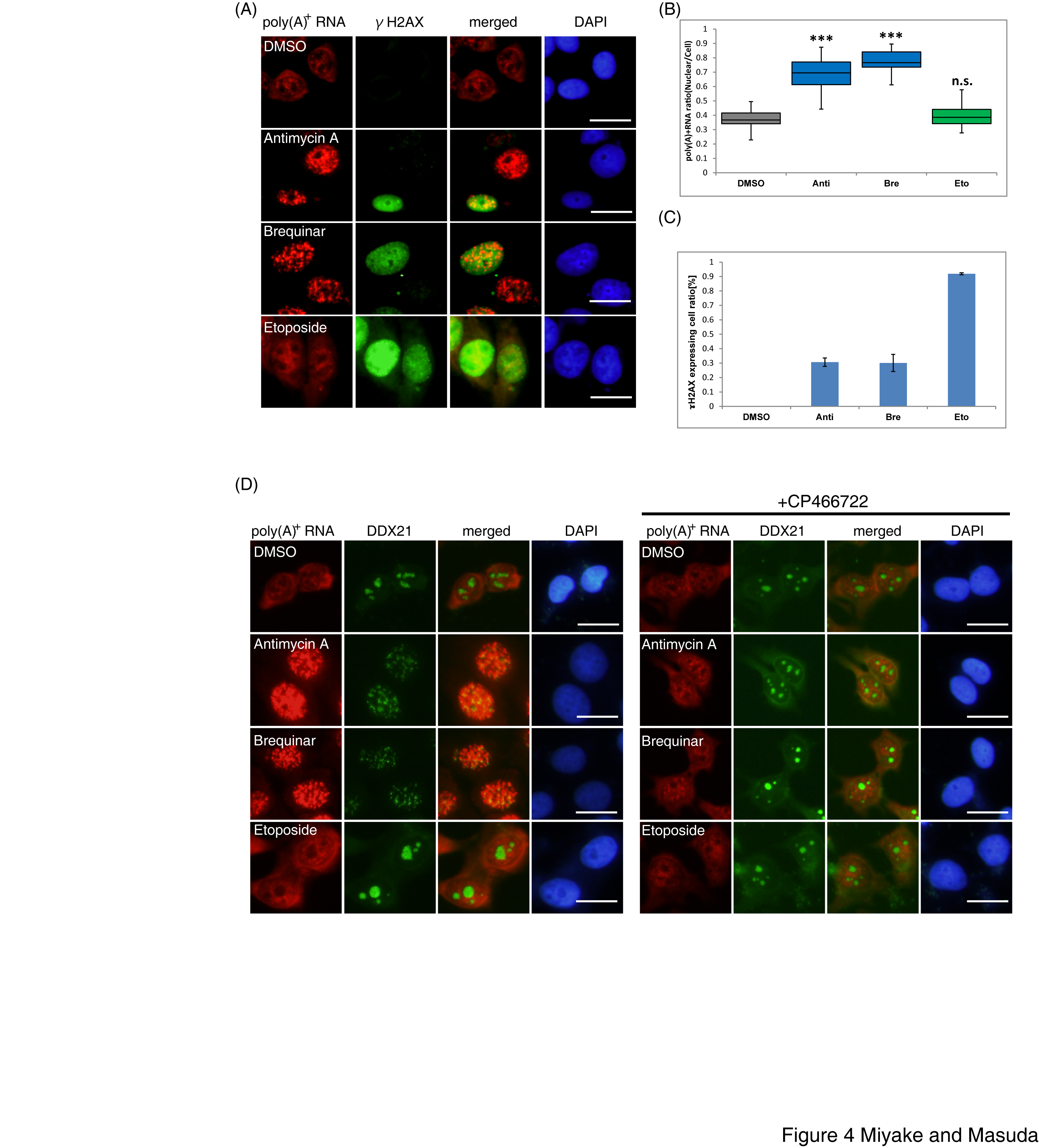

Supplement: Supplemental Material [file KRNB_A_2146919_SM2131.zip › sup-Fig.4.tif]

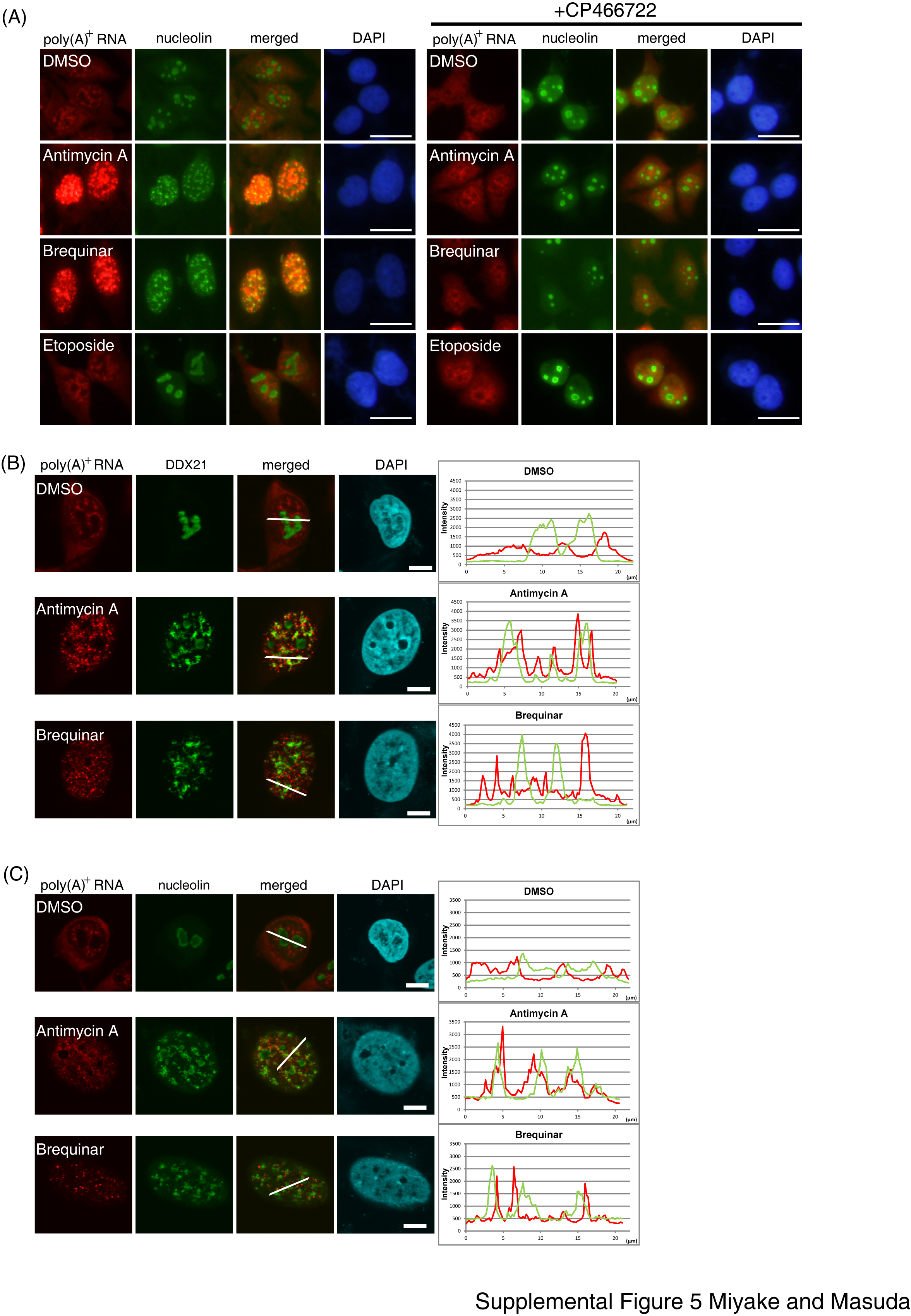

Supplement: Supplemental Material [file KRNB_A_2146919_SM2131.zip › sup-Fig.5.tif]

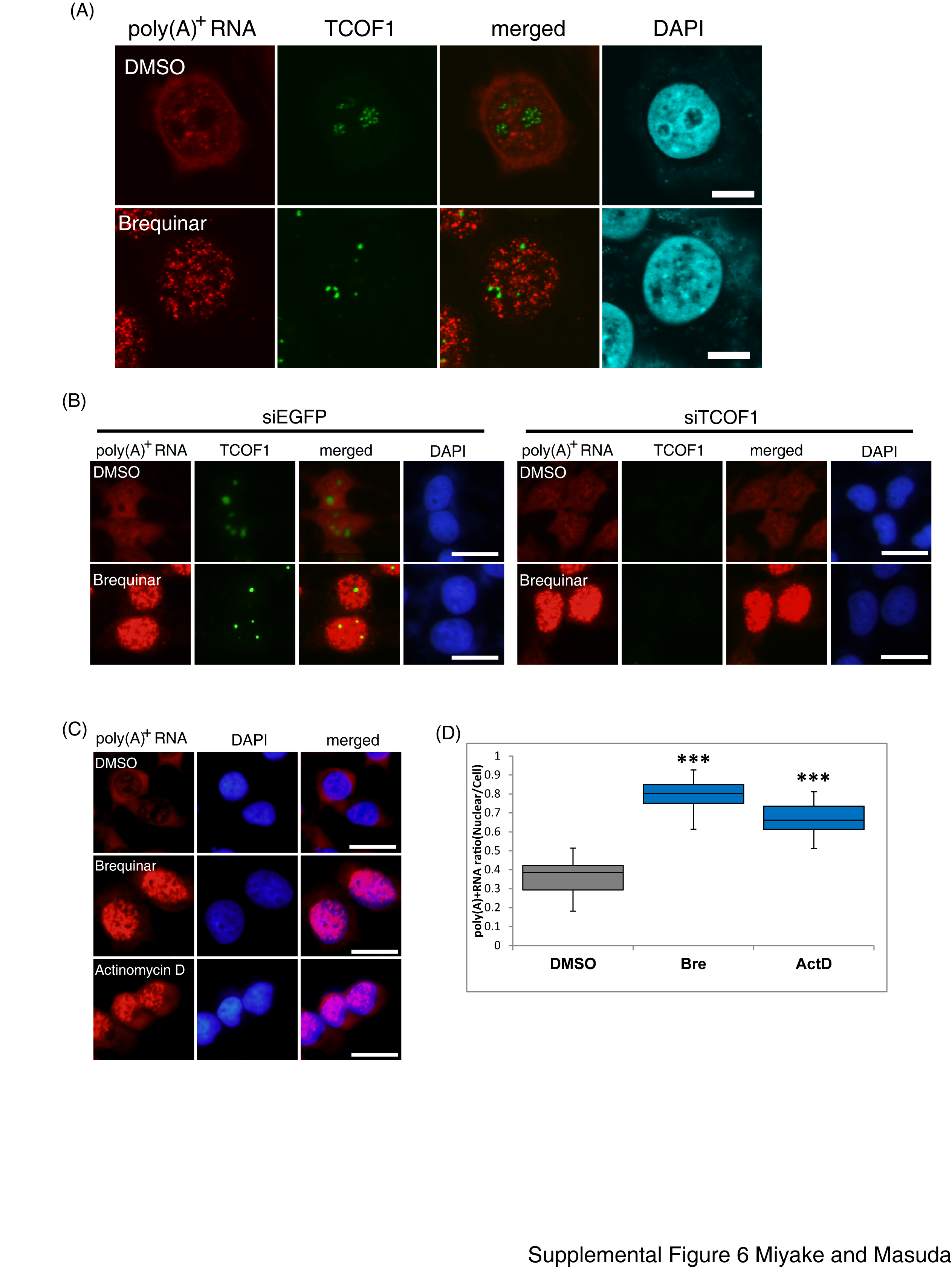

Supplement: Supplemental Material [file KRNB_A_2146919_SM2131.zip › sup-Fig.6.tif]
